# Parental Perceptions of Healthy Eating and Actual Nutrient Intake: Analysis of the Nutritional Status of Children Aged 1–6 Years in Urban Areas of Central Kazakhstan

**DOI:** 10.3390/ijerph23010109

**Published:** 2026-01-15

**Authors:** Svetlana Plyassovskaya, Yelena Pozdnyakova, Xeniya Mkhitaryan

**Affiliations:** 1 School of Public Health and Biomedicine, Karaganda Medical University, Karaganda 100000, Kazakhstan; 2 Department of Biomedicine, Karaganda Medical University, Karaganda 100000, Kazakhstan; 3 Department of Physiology, Karaganda Medical University, Karaganda 100000, Kazakhstan

**Keywords:** preschool children, dietary intake, parental perceptions, nutrient deficiencies, Central Kazakhstan

## Abstract

**Highlights:**

**Public health relevance—how does this work relate to a public health issue?**

- This study provides detailed, age-stratified data on actual macro- and micronutrient intakes in urban children aged 1–6 years in Central Kazakhstan, a region where early-childhood nutrition is critical but remains poorly documented.
- We show that many apparently healthy preschoolers have diets with systematic deficits in fats, dietary fiber, vitamin C, calcium, iron, and energy (in specific age groups), alongside persistent excess sodium, highlighting an under-recognized form of nutritional risk.

**Public health significance—why is this work of significance to public health?**

- The results identify younger preschoolers (especially 3–4-year-olds entering kindergarten) as the most vulnerable subgroup, with clustering of multiple macro- and micronutrient shortfalls that may affect growth and future noncommunicable disease risk.
- Weak, non-significant correlations between parental dietary priorities and children’s actual nutrient intakes reveal a perception–intake gap, suggesting that current public messages about “healthy eating” do not effectively translate into nutritionally adequate diets for young children.

**Public health implications—what are the key implications or messages for practitioners, policy makers and/or researchers in public health?**

- Public health programs and preschool feeding policies in Central Asia should move beyond a primary focus on “avoiding unhealthy foods” and explicitly promote adequate intake of fats, dietary fiber, vitamin C, calcium, and bioavailable iron in early-childhood diets, while reducing sodium.
- The age-specific patterns we document can be used to target counseling, menu revision and nutritional surveillance toward families and preschool institutions where multiple nutrient deficits are most likely, particularly in children aged 3–4 years.

**Abstract:**

Parental perceptions of healthy eating often diverge from children’s actual diets, but this gap is poorly documented in Central Asia. We examined how parents’ priorities for key food groups relate to nutrient intakes in 390 urban children aged 1–6 years in Central Kazakhstan. In a cross-sectional study, parents completed a 24 h multiple-pass dietary recall and rated the importance of fats and sweets, meat and fish, dairy, vegetables and fruits, and bread and potatoes on 5-point scales. Nutrient intakes were calculated using software, compared with national DRIs, and analyzed using rank-based tests and Spearman correlations. Parents reported near-ceiling priority for restricting fats and sweets and consistently high priority for bread and potatoes, whereas vegetables, fruits, meat/fish, and dairy were rated moderately important, with dairy under-prioritized in 1–2-year-olds. On the recalled day, median intakes of fat, dietary fiber, vitamin C, and calcium were below national recommendations at all ages, and median intakes of iron, thiamine, and niacin were particularly low at 3–4 years, while sodium intake exceeded recommended levels; the 3–4-year group showed the most pronounced clustering of shortfalls. Prevalence estimates indicated that most children had intakes below recommendations for dietary fiber and calcium and above recommendations for sodium, underscoring population-wide nutritional imbalance. Across all scales, parental priorities showed only weak, non-significant associations with nutrient intakes (|r| < 0.11). These findings indicate a perception–intake gap and support interventions that ensure adequate fats, fiber, vitamin C, calcium, and bioavailable iron in preschool diets.

## 1. Introduction

Nutrition in early and preschool childhood is currently regarded as one of the key determinants of health across the life course [1]. Classical and contemporary work in evidence-based nutrition shows that deficits or excesses of energy and specific nutrients during “critical windows” of development are associated not only with stunting and delays in cognitive development, but also with an increased risk of metabolic syndrome, cardiovascular disease, and other noncommunicable diseases in adulthood [2,3]. It is precisely in the first years of life that eating habits, taste preferences, and patterns of eating behavior are formed; these are then consolidated and determine the risk of both deficiency states (anemia, hypovitaminosis, rickets) and obesity and related metabolic disorders [4,5].

International recommendations emphasize that after a period of exclusive breastfeeding during the first 6 months of life, qualitatively and quantitatively adequate complementary feeding becomes particularly important, providing the child with sufficient amounts of protein, fats, carbohydrates, dietary fiber, vitamins, and minerals while limiting free sugars, saturated fats, and trans fats [6,7]. However, numerous reports and reviews show that in many countries of the world, young children receive diets that lack diversity, are poor in vegetables and fruits and in iron- and zinc-rich animal-source foods, while the consumption of industrially processed products, sugary drinks, and fast food is increasing [8,9,10].

The situation in Eastern Europe and Central Asia is of particular concern. According to a regional landscape analysis by UNICEF, a troubling situation persists: along with ongoing stunting in a subset of children, there are significant rates of anemia and a rapidly growing prevalence of overweight and obesity in preschool and school-aged children [11]. The document emphasizes that the countries of Central Asia (Kazakhstan, Kyrgyzstan, Tajikistan, Turkmenistan, Uzbekistan) have made notable progress in reducing stunting and improving indicators of breastfeeding, but still face low dietary diversity, seasonal limitations in the availability of fresh vegetables and fruits, a high share of foods containing trans fats, and insufficiently effective counseling on child nutrition. In the cities of Central Asia, the availability of energy-dense but nutritionally “empty” foods (cookies, sweet pastries, sugary drinks, street fast food) is increasing faster than the availability of fresh, minimally processed foods, which leads to a shift in diet structure toward sugars and refined carbohydrates [12].

According to the Global Nutrition Report and related national profiles, the countries of Central Asia are, in general, approaching target indicators for reducing stunting and wasting, but still have substantial problems with anemia, micronutrient deficiencies, and increasing overweight [13]. Kazakhstan, according to the same source and UNICEF data, is characterized by a comparatively low prevalence of stunting and wasting among children under 5 years of age, but a persistent burden of anemia and other manifestations of hidden hunger [14,15]. Individual studies indicate a high prevalence of vitamin D, iron, and vitamin A deficiency in children of various age groups, which confirms the relevance of assessing not only physical development status but also actual intake of key micronutrients [16,17].

Kazakhstan is classified as an upper-middle-income country, but marked regional and social inequalities persist, including higher poverty and material deprivation among large, low-income and rural families. Recent UNICEF assessments show that children remain more vulnerable to poverty than adults, and that multidimensional deprivation continues to affect access to quality food, health care, and early-childhood services, despite overall macroeconomic growth and investments in social protection [18,19,20,21].

At the same time, Kazakhstan is undergoing a rapid “nutrition transition” with increasing availability of ultra-processed products, sugar-sweetened beverages, refined wheat products, and energy-dense snacks, especially in urban areas. Household survey data indicate a gradual shift from traditional diets based on home-cooked cereal and meat dishes toward patterns with higher intakes of fats, sugars, and processed foods, which contributes to the emerging double burden of malnutrition (coexistence of micronutrient deficiencies with overweight and obesity). These changes are shaped by urbanization, aggressive food marketing, the expansion of modern retail and food-service outlets, as well as cultural norms that favor large portions of meat, white bread, and sweet tea during family meals and celebrations [22,23,24].

Beyond individual households, children’s diets in Kazakhstan are also shaped by the local food environment and the organization of preschool and school feeding. UNICEF’s national Situation Analysis highlights marked socio-economic and regional inequalities in child poverty and in access to early childhood education services, which translate into heterogeneous opportunities for healthy eating between major cities and other regions [25]. Recent evaluations of school meals conducted by UNICEF and the National Healthy Nutrition Center in several regions, together with global surveys of Kazakhstan’s school feeding system, show that while kindergarten and school canteens can provide important sources of regular hot meals, there remain challenges related to menu quality, alignment with nutritional standards, and equity of coverage between schools and districts [26,27,28].

For neighboring countries of Central Asia, a substantial body of data has been accumulated on breastfeeding practices, complementary feeding, and the diets of children aged 6–23 months, demonstrating a systematic mismatch between actual feeding practices and international recommendations. In Tajikistan, according to the Demographic and Health Survey and formative research, only about one-third to forty percent of children aged 6–23 months receive minimum dietary diversity and adequate meal frequency, whereas the early introduction of sweet cookies and drinks and delayed introduction of animal-source foods into the diet are common [29]. In Kyrgyzstan, an intervention study involving counseling mothers on infant and young child feeding showed that even relatively simple and low-cost measures can improve consumption of vitamin A-rich foods, eggs, and vegetables among children aged 6–23 months, but baseline dietary diversity remains low [30]. Studies on the use of behavioral insights in Kyrgyzstan also show that children’s food preferences and diets are closely linked to cultural norms, intra-household resource allocation, and parental awareness [31].

Thus, for the Central Asian region as a whole, it has been shown that during the period from 6 months to 2 years, children often receive neither sufficient energy nor an adequate range of foods, and diet structures are characterized by a low share of vegetables and fruits; lacking sources of iron, zinc, and vitamin C; and, at the same time, a growing role of processed foods rich in sugar and trans fats [32,33]. However, most of these data relate either to children aged 6–23 months or to vulnerable groups (children in residential institutions, rural populations, families living in poverty). For children older than 2–3 years, especially those attending preschool institutions in cities, information on actual macro- and micronutrient intake is much more limited.

Another underexplored aspect concerns the discrepancy between parents’ stated attitudes toward healthy eating and the actual structure of children’s diets. International studies show that parents often overestimate the quality of their children’s diet, focusing on restricting obviously “unhealthy” foods (sweets, fast food, soft drinks), while underestimating the importance of adequate intake of fats, vegetables and fruits, sources of dietary fiber, and vitamin C, as well as foods rich in iron and zinc [34,35,36]. For the countries of Central Asia, and especially for Kazakhstan, this “perception gap” has been virtually unexplored. In Kazakhstan, studies of child nutrition most often address specific aspects: vitamin D deficiency and rickets, anemia, the status of children in residential institutions, or general changes in dietary patterns of the population in the context of the “nutrition transition” [37,38,39,40].

Data on the actual macro- and micronutrient profile of the diets of healthy urban children aged 1–6 years are extremely limited, and comparisons of these diets with national standards and international recommendations are practically absent. At the population level, there is also a lack of detailed information on how parental attitudes (for example, efforts to limit fats and sweets or attitudes toward vegetables, fruits, dairy, and meat products) relate to children’s actual intake of energy, protein, fats, carbohydrates, dietary fiber, vitamins, and minerals.

The aim of this study was a comprehensive assessment of the actual diets of young and preschool children (aged 1–6 years) in urban settings of Central Kazakhstan using a 24 h dietary recall; determination of the degree to which intake of major macro- and micronutrients complies with the national recommendations of the Republic of Kazakhstan; and analysis of the relationship between this intake and parental priorities regarding key food groups.

## 2. Materials and Methods

### 2.1. Study Design

The study was conducted as a descriptive cross-sectional observational study. Data were collected by the authors themselves from 9 October 2023 to 1 October 2024 using a standardized protocol for a 24-h dietary recall administered to parents/legal guardians. Before the field phase, all interviewers received uniform training on interview techniques, the use of supporting materials (portion-size photographic atlases, measuring utensils), and data entry into electronic forms. No interventions in children’s usual diets were undertaken; actual intake over the 24 h preceding the interview was recorded, in combination with an assessment of parental dietary priorities using standardized scales (score-based ratings of the perceived importance of major food groups). The full wording of the parent-administered questionnaire is provided in the Supplementary Materials (Questionnaire File S1). In addition to dietary data, the parent-administered questionnaire collected basic sociodemographic information, including child’s sex and age, parental age, family structure, parental education and self-reported household income category.

### 2.2. Object and Study Population

The present analysis is based on data from a study database compiled across participating facilities of 24-h dietary recalls collected in urban outpatient healthcare facilities and kindergartens in two major cities of Kazakhstan (Karaganda and Astana). For the purposes of this study, all children aged 1–6 years for whom at least partially completed 24-h dietary recalls were available in the database were initially selected (*n* = 545 unique children) (Table 1).

The underlying survey used a facility-based, non-probability sampling strategy. In each participating urban outpatient clinic and kindergarten in Karaganda and Astana, fieldworkers approached all children aged 1–6 years who attended the clinic for routine visits or were present in the kindergarten on survey days, provided that a parent or legal guardian was available and willing to be interviewed. Thus, within each facility the recruitment approximated consecutive enrolment of all eligible children during the fieldwork period, and the present analysis includes the full subset of 1–6-year-olds with available 24-h recalls from this facility-based sample.

**Figure 1:**
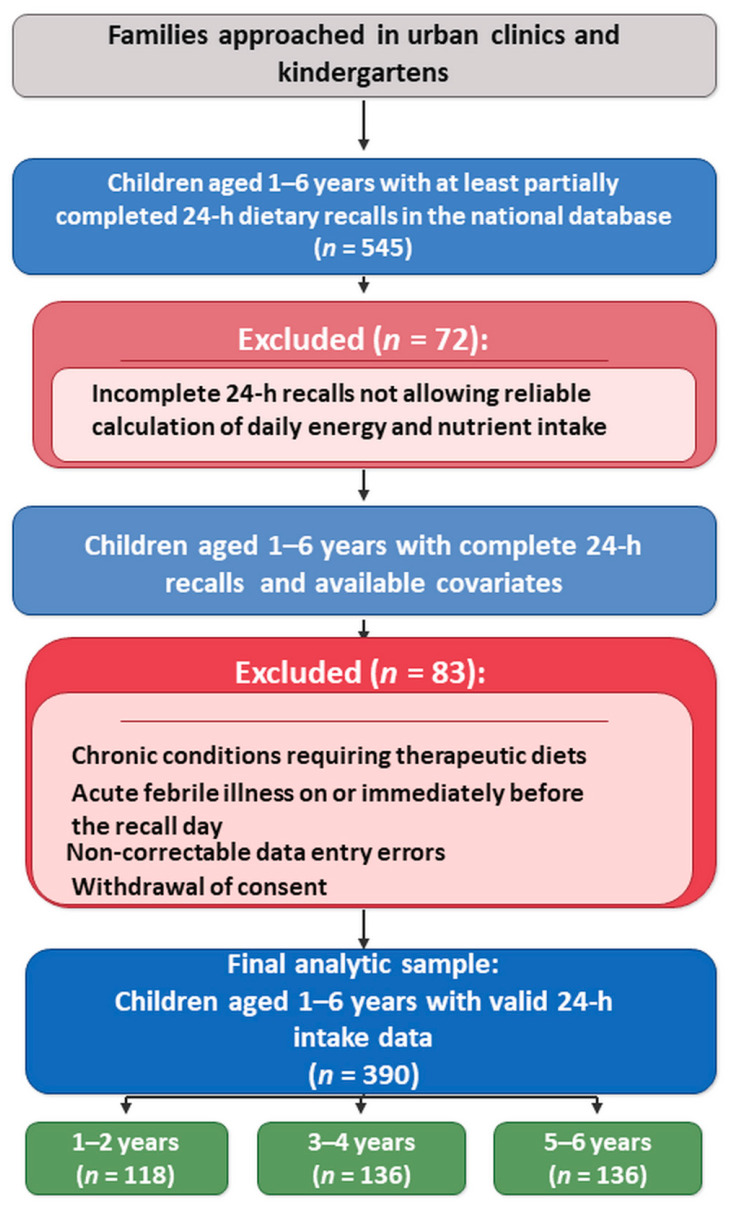
Participant flow for children aged 1–6 years.

From the initial sample, 72 children with incomplete 24-h dietary recalls that did not allow reliable estimation of daily energy and nutrient intake were excluded. After application of the predefined exclusion criteria, an additional 83 children were excluded. The final analytical sample comprised 390 children (1–2 years: *n* = 118; 3–4 years: *n* = 136; 5–6 years: *n* = 136) (Figure 1).

Three age subgroups were included in the analysis:

1. 1–2 years (*n* = 118)—young children predominantly fed at home after cessation of breastfeeding (occasional episodes of mixed feeding without predominance of breast milk were allowed).
2. 3–4 years (*n* = 136)—younger preschool children attending kindergarten and receiving a substantial share of their daily diet through an organized feeding system (preschool institution menus), while maintaining family meals at home.
3. 5–6 years (*n* = 136)—older preschool children who, as a rule, attend kindergarten regularly and have greater autonomy in their eating behavior (choice of foods at home and in organized feeding settings).

The analytical sample included 390 children aged 1 to 6 years, with a slight predominance of girls. Overall, 41.8% of the children resided in Astana and 58.2% in Karaganda, and the majority spent the day in kindergarten. The vast majority lived with married or cohabiting parents; maternal educational attainment was generally high, and household income predominantly fell into the middle and higher categories (Table 2).

Children were stratified into three age subgroups: 1–2 years, 3–4 years, and 5–6 years. This choice of intervals reflects key stages in the formation of the diet: the period of cessation of breastfeeding and transition to the family table (1–2 years), early preschool age with predominantly family-based feeding (3–4 years), and older preschool age with greater child autonomy and regular attendance of preschool institutions (5–6 years).

To compare actual diets with reference values, we used the national recommendations of the Republic of Kazakhstan on nutrition for children aged 1–3 and 4–6 years [30].

### 2.3. Eligibility Criteria

Inclusion Criteria

Children were included in the study if the following conditions were met:

- age 12–35 months (1–2-year group), 36–59 months (3–4-year group), or 60–83 months (5–6-year group);
- co-residence with a parent/legal guardian able to provide reliable information on the child’s actual diet over the previous 24 h;
- signed informed consent from the parent/guardian for the child’s participation in the study;
- permanent residence in the city (no prolonged trips to rural areas during the week preceding the interview).

Exclusion Criteria

Children were excluded from the study if at least one of the following was present:

- medical conditions requiring an individualized therapeutic diet (celiac disease, phenylketonuria, severe allergic diseases necessitating mandatory exclusion diets, etc.) as reported by parents;
- acute illnesses with fever on the day preceding the interview or on the interview day;
- incomplete dietary questionnaires (observation period <24 h or absence of one/several key meals);
- obvious data entry errors that could not be corrected at the verification stage (for example, total energy <300 or >3500 kcal/day for the given age, logically impossible combinations of portions and dishes);
- parent/guardian refusal to continue participation or withdrawal of informed consent at any stage.

### 2.4. Ethical Considerations

The study complied with the principles of the Declaration of Helsinki. Before inclusion, parents/guardians were provided complete information on objectives, procedures, risks/benefits, and confidentiality; written informed consent was obtained. The study protocol was approved by the Ethics Committee of Karaganda Medical University (protocol No. 1 of 18 August 2023).

### 2.5. Dietary Assessment

Dietary information was obtained using a 24-h dietary recall in a multiple-pass interview format: listing all eating occasions, detailed reconstruction of dishes and ingredients, cooking methods, portion-size estimation (with visual aids and measuring utensils), a probing pass, and a final review. To minimize systematic error, interviews were distributed across days of the week; holidays were excluded where possible. In households, interviews were conducted with the primary feeding adult; for children aged 3–4 years, all eating occasions over the 24-h period were taken into account, including meals in kindergarten (according to preschool menus/food production cards and/or the caregiver’s report).

For children who attended kindergarten on the recall day (mainly 3–4- and 5–6-year-olds), intake at institutional meals was estimated using standard menus and production (technical) cards combined with semi-quantitative caregiver reports. For each dish, the standard portion size (g) from the production card was multiplied by an approximate consumption factor based on the caregiver’s evaluation (“ate all or almost all”, “about half”, “only tasted/ate little”, “refused”), corresponding to fractions of 1.0, 0.5, 0.25 and 0, respectively. The resulting gram amounts were entered into the nutrient calculation software in the same way as home foods reported by parents.

The chemical composition and energy value of the diet were analyzed using the software package “Program for calculating the nutritional and biological value of the average daily diet” [41,42,43].

### 2.6. Statistical Analysis

Statistical analysis was performed in GraphPad Prism 8.0 (GraphPad Software, USA). For all continuous dietary variables (daily intake of energy, macro- and micronutrients), we calculated the median, interquartile range (Q1–Q3), minimum, and maximum for each age subgroup (1–2, 3–4, and 5–6 years).

Normality was assessed using the Anderson–Darling, D’Agostino–Pearson, Shapiro–Wilk, and Kolmogorov–Smirnov tests. Given the systematic deviation from a normal distribution for most variables in all age groups, nonparametric methods were applied.

To compare the three age groups (1–2, 3–4, and 5–6 years) on continuous parameters (daily intake of proteins, fats, carbohydrates, dietary fiber, vitamins, minerals, and total energy intake), we used the two-sided Kruskal–Wallis test. When the global result was statistically significant, we conducted post hoc analysis using Dunn’s test with adjustment for multiple comparisons. In the results, we reported the H statistic, the *p* value, and the pairs of age subgroups between which statistically significant differences were identified (adjusted *p* values).

Given the descriptive and exploratory nature of this study, one-sample non-parametric tests comparing median intakes with national reference values were performed for a broad panel of nutrients. A priori, particular emphasis was placed on energy, total fat, dietary fiber, calcium, iron, vitamin C and sodium as key nutrients of public health relevance, while analyses for other micronutrients were considered exploratory. No formal multiplicity correction was applied across all nutrients, and *p*-values should therefore be interpreted with caution, with primary weight given to the magnitude and consistency of observed differences from reference values.

To assess deviations of actual daily nutrient intake from the physiological reference values established in the national recommendations of the Republic of Kazakhstan (age-specific requirements for energy and nutrients), we applied the two-sided one-sample Wilcoxon test within each age subgroup. Actual medians were compared with the corresponding reference values for the given age category; in the results, we reported the median of actual intake, the reference value, and the *p* value of the test. For interpretation, we additionally calculated the relative deviation in percent (% above or below the national reference).

To examine the relationship between parental attitudes toward different food groups and children’s actual nutrient intake, we used Spearman rank correlation. Score-based ratings on five-point scales of dietary priorities (fats and sweets, meat and fish, dairy products, vegetables and fruits, bread and potatoes) were treated as ordinal predictors. For each scale, physiologically relevant outcomes were preselected (for example, priority for dairy products—calcium, riboflavin, protein; priority for vegetables and fruits—dietary fiber, vitamin C, potassium; intended restriction of “fats and sweets”—lipids, energy intake, sodium, etc.). Spearman rank correlation coefficients (Spearman r) were calculated in the pooled sample of children aged 1–6 years. In the results, we reported r, the 95% confidence interval, and the two-sided *p* value; the sign of r was interpreted as the direction of the association, and its absolute value as the strength of the association under a monotonic relationship.

Correlations between parental dietary priority scores and children’s nutrient intakes were estimated using Spearman’s rank correlation in the pooled sample of 1–6-year-olds. Given the descriptive and exploratory aims and the limited sample sizes within individual age–city strata, these correlation analyses were performed without formal adjustment for potential confounders such as age group or city of residence and should be interpreted as hypothesis-generating.

### 2.7. Data Quality Management

All questionnaires underwent double verification: logical checks (sequence of eating occasions, total energy within acceptable age-specific ranges) and control of nutrient outliers (visual inspection of box plots, repeat interview in case of anomalies, in agreement with the parent). For kindergarten menus, we used standardized recipe cards/technological cards and/or daily portion reports approved by the administration of the institution.

## 3. Results

### 3.1. Parental Priorities Regarding Major Food Groups in the Diets of Children Aged 1–6 Years

To assess awareness of healthy eating principles, parents rated major food groups on a 1–5 scale, where 1 indicated the group that should predominate in the child’s diet and 5 the group that should be minimized. In reporting, we refer to scores for core food groups as priority (perceived importance) ratings, whereas the “Fats and sweets” scores are interpreted as an intended restriction rating.

#### 3.1.1. Fats and Sweets

On the “Fats and sweets” scale, ratings in all age subgroups were strongly skewed toward the upper end of the scale: the median was 5.0 [25th; 75th percentiles: 5.0; 5.0], with a minimum of 1 and a maximum of 5; mean values were also close to the maximum (4.66 ± 0.75 points in the 1–2-year group, 4.67 ± 0.89 points in the 3–4-year group, and 4.54 ± 1.03 points in the 5–6-year group). The Kruskal–Wallis test did not reveal statistically significant differences between ages (H = 2.03; *p* = 0.36), and pairwise Dunn comparisons likewise showed no significant contrasts (1–2 vs. 3–4 years: *p* = 0.864; 1–2 vs. 5–6 years: *p* > 0.9999; 3–4 vs. 5–6 years: *p* = 0.537). This indicates that, regardless of the child’s age, parents almost unanimously declare a maximally pronounced intention to control the intake of foods high in fat and sugar (Table 3).

#### 3.1.2. Meat and Fish

When evaluating meat and fish products, the median score in all age groups was the same, 2.0 points (1.0; 3.0), with a minimum of 1 and a maximum of 5; mean values were also almost identical (2.13 ± 1.04 points at 1–2 years, 2.21 ± 1.14 points at 3–4 and 5–6 years). The nonparametric one-way analysis (H = 0.23; *p* = 0.891) and Dunn’s test (adjusted *p* > 0.9999 for all age-group pairs) confirmed the absence of statistically significant differences between subgroups. Thus, parents perceive meat and fish as a moderately prioritized, but not dominant, part of the diet, and this position remains stable throughout the preschool period.

#### 3.1.3. Dairy Products

For dairy products, the median score was also 2.0 points in all subgroups; however, the distributions showed a pronounced age-related pattern. At 1–2 years, the interquartile range was narrower (1.0; 2.25), with a maximum of 4 points, whereas at 3–4 and 5–6 years, the range widened to (1.0; 3.0) with a maximum of 5 points. Mean values increased consistently from 1.85 ± 0.81 points in the 1–2-year group to 2.27 ± 1.08 at 3–4 years and 2.28 ± 1.09 at 5–6 years. The Kruskal–Wallis test revealed significant age differences (H = 11.90; *p* = 0.0026), and Dunn’s post hoc analysis showed that it was specifically the 1–2-year group that differed significantly from both older groups (1–2 vs. 3–4 years: *p* = 0.0080; 1–2 vs. 5–6 years: *p* = 0.0071), whereas scores at 3–4 and 5–6 years did not differ (*p* > 0.9999). This indicates that in parents’ eyes the priority of dairy products is lowest at the youngest age and increases markedly by age 3, after which it remains relatively stable through age 6.

#### 3.1.4. Vegetables and Fruits

For vegetables and fruits, the median score was the same across all three age subgroups, at 3.0 points (2.0; 4.0); the minimum score remained 1 point, while the maximum reached 5 points in the 1–2- and 5–6-year groups and 4 points in the 3–4-year group. Mean values showed a moderately nonlinear trajectory: 2.91 ± 1.22 points at 1–2 years, decreasing to 2.56 ± 1.14 at 3–4 years and then increasing again to 2.79 ± 1.18 at 5–6 years. The one-way Kruskal–Wallis analysis yielded a borderline *p* value (H = 5.88; *p* = 0.0529), and pairwise Dunn comparisons did not identify statistically significant differences (minimum adjusted *p* = 0.0524 for the 1–2 vs. 3–4-year pair; for the remaining pairs, *p* > 0.35). Taken together, this allows us to characterize parental attitudes toward vegetables and fruits as a “moderate priority” without statistically confirmed age shifts, with only a tendency toward a temporary decrease in importance at 3–4 years and partial recovery by 5–6 years.

#### 3.1.5. Bread and Potatoes

Bread and potatoes were viewed across all age subgroups as a consistently important “base” of the diet. Median scores were high: 3.5 points [3.0; 4.0] at 1–2 years, 3.0 (2.0; 4.0) at 3–4 years, and 4.0 (2.0; 4.0) at 5–6 years; minimum scores were 1 point and maximum scores 5 points (response range 1–5). Mean values were almost identical across ages (3.36 ± 1.15; 3.24 ± 1.03; and 3.24 ± 1.12 points, respectively). The nonparametric one-way analysis (H = 1.13; *p* = 0.569) and Dunn’s post hoc test (1–2 vs. 3–4 years: *p* = 0.9074; 1–2 vs. 5–6 years and 3–4 vs. 5–6 years: *p* > 0.9999) confirmed the absence of statistically significant age differences. Thus, bread and potatoes are consistently perceived by parents as a stably important, basic component of the child’s diet at all ages from 1 to 6 years.

Overall, the structure of parental priorities is as follows: a maximally strict declared control over fats and sweets, a consistently high basic status of bread and potatoes, a moderate and only slightly changing priority for meat and fish products, a “catch-up” increase in the importance of dairy products after age 2, and only a moderate, non-dominant priority for vegetables and fruits.

### 3.2. Characteristics of Actual Dietary Intake (Macronutrients)

In all age subgroups, the distributions of macronutrient intake deviated from normality; therefore, nonparametric tests were used for evaluation.

#### 3.2.1. Proteins

In children aged 1–2 years, the median protein intake is 35.80 g/day (IQR 21.73–53.41 g; range 11.71–96.30 g) while the recommended level for this age group is 30.0 g/day, that is, the median is 5.80 g/day (19%) above the recommended level. The one-sample Wilcoxon test shows that this excess is statistically significant (*p* = 0.0010). The very wide spread of values indicates high interindividual variability of the protein component of the diet.

At 3–4 years of age, the median protein intake is 38.87 g/day (IQR 29.63–58.25 g; range 13.66–157.8 g) whereas the recommended level for this age group is 50.0 g/day, that is, 11.13 g/day (22%) below the recommended level. The one-sample Wilcoxon test confirms a statistically significant protein deficit (*p* < 0.0001). The range of values in this group is the largest, reflecting extremely high interindividual variability: there are both children with a marked protein deficit and children with excessive intake.

In children aged 5–6 years, the median protein intake is 43.50 g/day (IQR 32.62–58.90 g; range 5.24–143.0 g) relative to the recommended level of 50.0 g/day for this age group, that is, 6.5 g/day (13%) below the recommended level. According to the Wilcoxon test, this difference does not reach statistical significance (*p* = 0.0534), which allows us to speak only of a trend toward insufficient protein intake. The spread of individual values remains wide, although somewhat smaller than in the 3–4-year group (Table 4).

The global Kruskal–Wallis test with subsequent Dunn’s test showed that significant between-group differences are present only between the youngest and the oldest children: in the 5–6-year group, protein intake ranks are higher than in the 1–2-year group (*p* = 0.0119). Differences between 1–2 and 3–4 years (*p* = 0.3618), as well as between 3–4 and 5–6 years (*p* = 0.2025), are not statistically significant, which reflects a comparable level of protein intake in the second and third age subgroups.

Overall, for protein, some children aged 1–2 years show excessive intake relative to the recommended level, at 3–4 years a pronounced median deficit is observed, and by 5–6 years median values partially approach the age-specific reference values, with a wide range of individual values persisting in each age group.

#### 3.2.2. Carbohydrates

In children aged 1–2 years, the median carbohydrate intake is 169.4 g/day (IQR 130.4–237.0 g; range 57.45–436.8 g) while the recommended level for this age group is 159.0 g/day, that is, the median is 10.4 g/day (7%) above the recommended level. The one-sample Wilcoxon test shows a statistically significant excess (*p* = 0.0200) against the background of a very wide range of individual values.

At 3–4 years of age, the median carbohydrate intake is 177.0 g/day (IQR 139.7–216.8 g; range 97.43–574.8 g) whereas the recommended level for this age group is 266.0 g/day. Children of this age on average receive 88.9 g/day less carbohydrates (33% below the recommended level), and the one-sample Wilcoxon test reveals a highly significant deficit (*p* < 0.0001). The range of values in this group is the largest, indicating very heterogeneous carbohydrate coverage in the diet.

In children aged 5–6 years, the median carbohydrate intake is 196.1 g/day (IQR 162.8–242.8 g; range 52.28–525.1 g) with the same recommended level of 266.0 g/day for this age range; the median deficit is 69.9 g/day (26% below the recommended level). The Wilcoxon test confirms a statistically significant discrepancy with the recommendations (*p* < 0.0001), with a very wide range of individual values persisting (Table 4).

According to the Kruskal–Wallis test with subsequent Dunn’s test, statistically significant between-group differences were identified when comparing children 1–2 and 5–6 years (*p* = 0.0247), as well as 3–4 and 5–6 years (*p* = 0.0235), whereas differences between 1–2 and 3–4 years were not significant (*p* > 0.9999). The mean ranks of carbohydrate intake systematically increased from the youngest to the oldest group, reflecting an age-related increase in the absolute carbohydrate component of the diet.

Overall, in children aged 1–2 years, median carbohydrate intake slightly exceeds the recommended level, whereas starting from age 3 a pronounced deficit relative to age-specific recommendations is observed. By 5–6 years, this deficit is partially reduced but, on average, not fully compensated; at the same time, interindividual variability remains high in all age subgroups.

#### 3.2.3. Dietary Fiber

In children aged 1–2 years, the median dietary fiber intake is 9.30 g/day (IQR 7.15–12.66 g; range 3.30–27.15 g) while the recommended level for this age group is 19 g/day. The median deficit is −9.70 g/day (about 51% below the recommended level). The one-sample Wilcoxon test confirms the high statistical significance of this deficit (*p* < 0.0001).

In the 3–4-year group, the median fiber intake is 9.87 g/day (IQR 7.15–12.86 g; range 3.56–36.55 g), whereas the recommended level for this age group is 25 g/day. Actual intake corresponds to only about 39% of the recommended level (median deficit −15.13 g/day), and the discrepancy with the reference value is statistically significant (*p* < 0.0001).

In children aged 5–6 years, the median dietary fiber intake is 10.74 g/day (IQR 8.07–14.40 g; range 3.64–66.70 g) relative to the same recommended level of 25 g/day for this age range; the median deficit is −14.26 g/day (around 57% below the recommended level). The one-sample Wilcoxon test again shows a highly significant difference from the recommendations (*p* < 0.0001) (Table 4).

Comparison of the three age groups using the Kruskal–Wallis test and subsequent Dunn’s test did not reveal statistically significant between-group differences (*p* > 0.05 in all pairwise comparisons). The slight increase in the median from 1–2 to 5–6 years does not translate into a statistically robust age gradient: the actual level of fiber intake remains similar across all three ages.

#### 3.2.4. Lipids

In children aged 1–2 years, the median fat intake is 32.15 g/day (IQR 22.49–49.05 g; range 7.54–87.86 g) while the recommended level for this age group is 49.0 g/day, that is, the median shortfall is 16.86 g/day (34% below the recommended level). The one-sample Wilcoxon test shows that this deficit is statistically significant (*p* < 0.0001); the wide spread of values reflects marked interindividual variability in the fat component of the diet.

In the 3–4-year group, the median fat intake is 42.16 g/day (IQR 28.89–57.40 g; range 7.55–158.5 g) whereas the recommended level for this age group is 60.0 g/day. The shortfall is 17.84 g/day (30% below the recommended level) and is likewise statistically significant according to the Wilcoxon test (*p* < 0.0001). The range of values is maximal in this group, indicating the presence of both children with a pronounced fat deficit and children with very high fat intake.

In children aged 5–6 years, the median fat intake is 53.43 g/day (IQR 33.69–75.22 g; range 2.72–116.3 g) relative to the recommended level of 60.0 g/day for this age range, that is, the median shortfall is 6.57 g/day (11% below the recommended level). This difference is also statistically significant (*p* = 0.0239), although the range of individual values remains wide, decreasing somewhat compared with the 3–4-year group (Table 4).

According to the Kruskal–Wallis test (H = 26.37; *p* < 0.0001) and Dunn’s test, children aged 1–2 years consume significantly less fat than children aged 3–4 (*p* = 0.0315) and 5–6 years (*p* < 0.0001), and children aged 3–4 years consume less than those aged 5–6 years (*p* = 0.0080). The mean ranks systematically increase from the youngest to the oldest group, reflecting an age-related strengthening of the fat component of the diet.

Thus, at all ages a persistent median fat deficit relative to the recommended level is observed, most pronounced at 1–4 years. By 5–6 years, median intake partially approaches the recommended values; however, substantial interindividual differences persist within each subgroup.

#### 3.2.5. Total Energy Intake

In children aged 1–2 years, the median energy value of the diet is 1170 kcal/day (IQR 851.8–1648 kcal/day; range 461.5–2596 kcal) while the recommended energy intake for this age group is 1200 kcal/day. Actual intake is only 29.9 kcal/day (2.5%) below the recommended level. The one-sample Wilcoxon test did not reveal a statistically significant difference from the recommended level (*p* = 0.4593), which allows us to regard the average caloric value of the diets in this group as generally consistent with age-specific recommendations, despite the very large interindividual variability.

At 3–4 years of age, the median caloric value of the diet is 1299 kcal/day (IQR 1064–1546 kcal/day; range 634.9–3548 kcal) while the recommended energy intake for this age group is 1800 kcal/day. Children of this age on average receive 501 kcal/day less energy (about 28% below the recommended level), and this deficit is statistically significant (*p* < 0.0001). The wide range of values indicates the presence of both children with a pronounced energy deficit and children with very high daily caloric intake.

In children aged 5–6 years, the median daily caloric value reaches 1510 kcal/day (IQR 1182–1871 kcal/day; range 256–3418 kcal) relative to the recommended energy intake of 1800 kcal/day for this age range. The median deviation is −289.6 kcal/day (−16% relative to the recommended level), and according to the Wilcoxon test it is statistically significant (*p* < 0.0001), indicating a persistent energy deficit despite the tendency toward higher caloric intake compared with the 3–4-year group (Table 4).

According to the Kruskal–Wallis test and Dunn’s test, there are no statistically significant differences between the 1–2- and 3–4-year groups (*p* = 0.5614), whereas the caloric value of the diets in children aged 5–6 years is significantly higher than in children aged 1–2 (*p* = 0.0002) and 3–4 years (*p* = 0.0030). The mean ranks for caloric intake are lowest in the 1–2-year group, intermediate in the 3–4-year-olds, and highest in the 5–6-year-olds, reflecting an age-related increase in energy intake.

Overall, a pronounced and statistically significant median energy deficit relative to the recommended level is already observed after age three, and by 5–6 years caloric intake increases partially but on average remains below recommendations, with a very wide spread of individual diets in all age subgroups.

In addition to comparing median intake values with national reference standards, we further assessed the prevalence of inadequate and excessive intake of energy and macronutrients (Table 5).

Although mean energy intake among children aged 1–2 years was close to the recommended level, more than half of children in this age group had daily energy intake below the recommended norm. In older age groups, the prevalence of inadequate energy intake increased substantially, affecting approximately 81% of children aged 3–4 years and 71% of children aged 5–6 years.

A particularly unfavorable pattern was observed for dietary fiber intake: more than 90% of children across all age groups consumed less than the recommended amount, indicating a persistent population-wide deficit. In contrast, protein intake showed a mixed pattern: a substantial proportion of children aged 1–2 years exceeded recommended levels, whereas the majority of children aged 3–4 years consumed protein below the recommended intake.

### 3.3. Characteristics of Actual Vitamin Intake

In all age subgroups, the distributions of vitamin intake deviated from normality; therefore, nonparametric tests were used for evaluation.

#### 3.3.1. Thiamine (Vitamin B1)

In children aged 1–2 years, the median thiamine intake is 0.467 mg/day (IQR 0.333–0.687 mg; range 0.207–1.160 mg), while the recommended thiamine intake for this age group is 0.50 mg/day. The median deviation from the recommended level is small and amounts to −0.033 mg/day (7% below the recommended value). The one-sample Wilcoxon test did not reveal a statistically significant difference (*p* = 0.8543).

In the 3–4-year group, the median thiamine intake is 0.499 mg/day (IQR 0.382–0.692 mg; range 0.211–1.725 mg) relative to a recommended intake of 0.60 mg/day. The median deficit is 0.101 mg/day (17% below the recommended level), and this reduction is statistically significant according to the one-sample Wilcoxon test (*p* = 0.0012).

In children aged 5–6 years, the median thiamine intake is 0.613 mg/day (IQR 0.444–0.788 mg; range 0.104–1.893 mg) compared with a recommended intake of 0.60 mg/day for this age range. The median deviation from the recommended level is small and positive (+0.013 mg/day, +2%), and no statistically significant differences were found by the Wilcoxon test (*p* = 0.2714) (Table 6).

Between-group analysis using the Kruskal–Wallis test and Dunn’s test showed no significant differences between children aged 1–2 and 3–4 years (*p* = 0.8265), whereas the 5–6-year group had higher ranks of thiamine intake than 1–2-year-olds (*p* = 0.0011) and 3–4-year-olds (*p* = 0.0077). The mean ranks (140.8; 156.5; 192.2) reflect an age-related increase in the adequacy of thiamine intake in the diet.

Overall, in children aged 1–2 and 5–6 years, median thiamine intake on average is close to the recommended level, whereas at 3–4 years a statistically significant deficit is observed (17% below the recommended level). As we move from the youngest to the older age groups, the distribution shifts toward higher intake levels; however, pronounced interindividual variability persists within each subgroup.

#### 3.3.2. Riboflavin (Vitamin B2)

In children aged 1–2 years, the median riboflavin intake is 0.6520 mg/day (IQR 0.5120–0.9638 mg; range 0.2340–1.739 mg), while the recommended riboflavin intake for this age group is 0.5000 mg/day. The median excess above the target level is 0.1520 mg/day (30% higher than the recommended level) and is statistically significant according to the Wilcoxon test (*p* < 0.0001).

In the 3–4-year group, the median riboflavin intake is 0.6590 mg/day (IQR 0.5120–0.9025 mg; range 0.2530–3.128 mg) relative to a recommended intake of 0.6000 mg/day. The median excess is 0.0590 mg/day (10% above the recommended level), and the one-sample Wilcoxon test also confirms the statistical significance of this surplus (*p* = 0.0002).

In children aged 5–6 years, the median riboflavin intake is 0.7015 mg/day (IQR 0.5548–0.9440 mg; range 0.0720–6.524 mg), compared with a recommended intake of 0.6000 mg/day for this age range. The median excess is 0.1015 mg/day (17% above the recommended level) and is likewise statistically significant according to the Wilcoxon test (*p* < 0.0001) (Table 6).

According to the Kruskal–Wallis test and Dunn’s test, no statistically significant differences between ages were identified: comparisons of 1–2 and 3–4 years (*p* = 0.8265), 1–2 and 5–6 years (*p* = 0.5173), and 3–4 and 5–6 years (*p* = 0.9029) were all non-significant. The mean ranks increase moderately from the youngest to the oldest subgroup (156.7; 164.1; 176.4), indicating only a slight age-related tendency toward higher riboflavin intake.

Thus, in all three age subgroups, median riboflavin intake meets or moderately exceeds the age-specific recommended intake, with no statistically confirmed differences between ages and with a wide range of individual values.

#### 3.3.3. Niacin (Nicotinic Acid, Vitamin PP)

In children aged 1–2 years, the median niacin intake is 4.99 mg/day (IQR 3.08–8.83 mg; range 1.52–16.35 mg), while the recommended niacin intake for this age group is 6.0 mg/day. The median deficit is −1.01 mg/day (17% below the recommended level); however, the one-sample Wilcoxon test did not show a statistically significant difference (*p* = 0.8782).

In the 3–4-year group, the median niacin intake is 6.25 mg/day (IQR 4.33–8.70 mg; range 1.21–45.00 mg) relative to a recommended intake of 8.0 mg/day. The median deficit is −1.75 mg/day (22% below the recommended level), and this reduction is statistically significant (*p* < 0.0001). The range of values is maximal in this group, encompassing both very low and very high intake levels.

In children aged 5–6 years, the median niacin intake is 7.23 mg/day (IQR 4.71–9.77 mg; range 1.16–34.32 mg), while the recommended intake for this age range is 8.0 mg/day. The median deviation is −0.77 mg/day (10% below the recommended level), and no statistically significant difference from the recommendation was detected by the Wilcoxon test (*p* = 0.1746) (Table 6).

Between-group analysis using the Kruskal–Wallis test and Dunn’s test showed that differences between 1–2 and 3–4 years were not statistically significant (*p* = 0.2882), whereas children aged 5–6 years had higher ranks of niacin intake than 1–2-year-olds (*p* = 0.0083). Differences between 3–4 and 5–6 years did not reach significance (*p* = 0.3195). The mean ranks (140.8; 164.8; 183.9) indicate a gradual age-related increase in niacin intake.

Overall, in all age subgroups, median niacin intake is somewhat below the recommended level; however, a statistically confirmed pronounced deficit is found mainly in children aged 3–4 years. In children aged 1–2 and 5–6 years, median values are closer to the recommendations, while high interindividual variability is maintained.

#### 3.3.4. Vitamin C

In children aged 1–2 years, the median vitamin C intake is 16.27 mg/day (IQR 9.05–23.02 mg; range 0.86–57.30 mg), while the recommended vitamin C intake for this age group is 30.0 mg/day. The median deviation is −13.74 mg/day (46% below the recommended level), and this reduction is statistically significant according to the Wilcoxon test (*p* < 0.0001).

In the 3–4-year group, the median vitamin C intake is 15.75 mg/day (IQR 7.29–27.60 mg; range 0.45–68.05 mg), relative to a recommended intake of 30.0 mg/day. The median deficit is −14.25 mg/day (48% below the recommended level) and is likewise highly significant (*p* < 0.0001).

In children aged 5–6 years, the median vitamin C intake is 17.81 mg/day (IQR 7.70–31.82 mg; range 0–133.0 mg), with the same recommended intake of 30.0 mg/day. The median deviation is −12.19 mg/day (41% below the recommended level); the Wilcoxon test also shows a statistically significant deficit (*p* < 0.0001), despite the higher upper quartile and the presence of children with very high intake levels (Table 6).

Comparison of age subgroups using the Kruskal–Wallis test and Dunn’s test did not reveal statistically significant between-group differences: in all pairwise comparisons, adjusted *p* values exceeded 0.66. The mean ranks (157.7; 164.7; 175.4) increase slightly with age but without a statistically robust age gradient.

Thus, in all three age groups, median vitamin C intake is substantially and statistically significantly below the recommended level (by 40–50%), with no marked differences between ages and with a very wide range of individual values.

The analysis of prevalence estimates for vitamin intake confirmed and extended the findings based on median values. The highest prevalence of inadequate intake was observed for vitamin C: more than 70% of children in all age groups consumed less than the recommended intake (Table 7).

With respect to thiamine and niacin, the most pronounced prevalence of deficiency was observed among children aged 3–4 years, whereas riboflavin intake exceeded recommended levels in the majority of children across all age groups.

### 3.4. Characteristics of Actual Mineral Intake

In all three age subgroups, the distributions of mineral intake (iron, potassium, calcium, magnesium, sodium, phosphorus) differed statistically significantly from normal; therefore, nonparametric tests were used in all cases.

#### 3.4.1. Iron (Fe)

In children aged 1–2 years, the median iron intake is 7.17 mg/day (IQR 4.56–10.06 mg; range 2.86–15.73 mg), while the recommended iron intake for this age group is 10.0 mg/day. The median deficit is 2.83 mg/day (28% below the recommended level), and this reduction is highly significant according to the Wilcoxon test (*p* < 0.0001).

In the 3–4-year group, the median iron intake is 8.18 mg/day (IQR 6.81–10.36 mg; range 2.57–32.20 mg), relative to the same recommended intake of 10.0 mg/day. The median deficit is 1.82 mg/day (18% below the recommended level) and is also statistically significant (*p* < 0.0001).

In children aged 5–6 years, the median iron intake reaches 9.73 mg/day (IQR 7.76–12.19 mg; range 1.24–48.60 mg), with the same recommended intake of 10.0 mg/day. The median deviation from the recommended level is small (−0.27 mg/day, 3% below) and is not statistically significant according to the Wilcoxon test (*p* = 0.7801) (Table 8).

According to the Kruskal–Wallis test and Dunn’s test, differences between 1–2 and 3–4 years are not statistically significant (*p* = 0.0679), whereas the 5–6-year group has substantially higher ranks of iron intake compared with 1–2-year-olds (*p* < 0.0001) and 3–4-year-olds (*p* = 0.0045). The mean ranks (126.4; 159.2; 196.8) reflect an age-related increase in iron adequacy.

Overall, in children aged 1–2 and 3–4 years, a pronounced and statistically significant median iron deficit relative to the recommended level is observed, whereas by 5–6 years median values approach the recommendations, although considerable interindividual variability persists within each age subgroup.

#### 3.4.2. Potassium (K)

In children aged 1–2 years, the median potassium intake is 1244 mg/day (IQR 864.4–1821 mg; range 554.9–4301 mg), while the recommended potassium intake for this age group is 400 mg/day. The median excess is 844.1 mg/day, which is 211% above the recommended level; the one-sample Wilcoxon test shows that this excess is statistically significant (*p* < 0.0001).

In the 3–4-year group, the median potassium intake is 1285 mg/day (IQR 944.4–1728 mg; range 445.6–3671 mg), relative to the recommended intake of 600 mg/day. The median excess is 685.1 mg/day (114% above the recommended level), and this is also statistically significant (*p* < 0.0001).

In children aged 5–6 years, the median potassium intake reaches 1368 mg/day (IQR 1008–1932 mg; range 373.2–5097 mg), with the same recommended intake of 600 mg/day. The median excess is 767.8 mg/day (128% above the recommended level) and is statistically significant according to the Wilcoxon test (*p* < 0.0001) (Table 8).

According to the Kruskal–Wallis test and Dunn’s test, no statistically significant differences were found between age subgroups: comparisons of 1–2 and 3–4 years (*p* > 0.9999), 1–2 and 5–6 years (*p* = 0.4220), and 3–4 and 5–6 years (*p* = 0.7261) were all non-significant. The mean ranks (156.1; 163.5; 177.3) show only a tendency toward an age-related increase in potassium intake.

Overall, in all three age subgroups, median potassium intake substantially and statistically significantly exceeds the recommended level, with a very wide range of individual values—from moderate to very high.

#### 3.4.3. Calcium (Ca)

In children aged 1–2 years, the median calcium intake is 360.7 mg/day (IQR 250.5–533.5 mg; range 108–1205 mg), while the recommended calcium intake for this age group is 700 mg/day. The median deficit is 339.3 mg/day (48% below the recommended level) and is statistically significant (*p* < 0.0001).

In the 3–4-year group, the median calcium intake is 372.1 mg/day (IQR 269.8–479.6 mg; range 140.4–1038 mg), relative to the recommended intake of 800 mg/day. The median deficit reaches 428.0 mg/day (54% below the recommended level) and is also statistically significant (*p* < 0.0001).

In children aged 5–6 years, the median calcium intake is 392.9 mg/day (IQR 289.4–523.1 mg; range 82–1205 mg), with the same recommended intake of 800 mg/day. The median deficit is 407.1 mg/day (51% below the recommended level) and is likewise statistically significant (*p* < 0.0001) (Table 8).

Between-group analysis using the Kruskal–Wallis test and Dunn’s test did not reveal statistically significant age differences: in all pairwise comparisons, adjusted *p* values exceeded 0.9999, and mean ranks (161.3; 164.3; 173.9) were similar.

Thus, in all three preschool age groups there is a deep and statistically significant median calcium deficit relative to the recommended level, with no signs of age-related improvement; a wide range of individual values persists in all subgroups.

#### 3.4.4. Magnesium (Mg)

In children aged 1–2 years, the median magnesium intake is 142.9 mg/day (IQR 100.1–207.7 mg; range 67.7–317.7 mg), while the recommended magnesium intake for this age group is 80.0 mg/day. The median excess is 62.9 mg/day (about 79% above the recommended level) and is statistically significant according to the Wilcoxon test (*p* < 0.0001).

In the 3–4-year group, the median magnesium intake is 158.5 mg/day (IQR 119.2–187.9 mg; range 54.6–610.3 mg), relative to the recommended intake of 100.0 mg/day. The median excess is 58.45 mg/day (about 58% above the recommended level), and it is highly significant according to the Wilcoxon test (*p* < 0.0001).

In children aged 5–6 years, the median magnesium intake reaches 164.3 mg/day (IQR 121.3–232.5 mg; range 37.6–921.0 mg), with the same recommended intake of 100.0 mg/day. The median excess is 64.31 mg/day (about 64% above the recommended level) and is also statistically significant (*p* < 0.0001), against the background of a very wide range of individual values (Table 8).

Between-group analysis using the Kruskal–Wallis test and Dunn’s test did not reveal statistically significant age differences: comparisons of 1–2 and 3–4 years (*p* = 0.99), 1–2 and 5–6 years (*p* = 0.076), and 3–4 and 5–6 years (*p* = 0.38) were non-significant. The mean ranks (149.1; 163.1; 181.3) indicate only a tendency toward an age-related increase in magnesium adequacy.

Overall, in all three age groups, a statistically significant median excess of magnesium intake relative to the recommended level is observed, with very high interindividual variability.

#### 3.4.5. Phosphorus

In children aged 1–2 years, the median phosphorus intake is 623.1 mg/day (IQR 455.3–823.9 mg; range 213.5–1341.0 mg), while the recommended phosphorus intake for this age group is 400.0 mg/day. The median excess is 223.1 mg/day (56% above the recommended level) and is statistically significant according to the Wilcoxon test (*p* < 0.0001).

In the 3–4-year group, the median phosphorus intake is 647.5 mg/day (IQR 525.8–807.6 mg; range 231.6–1798.0 mg), relative to the recommended intake of 500.0 mg/day. The median excess is 147.5 mg/day (30% above the recommended level) and is also statistically significant (*p* < 0.0001).

In children aged 5–6 years, the median phosphorus intake reaches 721.4 mg/day (IQR 575.7–915.1 mg; range 186.0–2493.0 mg), with the same recommended intake of 500.0 mg/day. The median excess is 221.4 mg/day (44% above the recommended level) and, according to the Wilcoxon test, is statistically significant (*p* < 0.0001) (Table 8).

According to the Kruskal–Wallis test and Dunn’s test, statistically significant between-group differences were observed only between children aged 1–2 and 5–6 years (*p* = 0.033), with the latter having higher mean ranks for phosphorus intake. Comparisons of 1–2 and 3–4 years (*p* = 0.86) and 3–4 and 5–6 years (*p* = 0.22) were not significant. The mean ranks (146.8; 162.1; 183.4) demonstrate a consistent tendency toward an age-related increase in phosphorus load.

Overall, in all three age subgroups, a statistically significant median excess of phosphorus intake relative to the recommended level is observed, with a very wide range of individual values and heterogeneous phosphorus adequacy.

#### 3.4.6. Sodium (Na)

In children aged 1–2 years, the median sodium intake is 1976 mg/day (IQR 1760–2810 mg; range 784.7–5923 mg), while the recommended sodium intake for this age group is 1000 mg/day. The median excess is 975.7 mg/day (98% above the recommended level), and the one-sample Wilcoxon test shows that this excess is highly statistically significant (*p* < 0.0001).

In the 3–4-year group, the median sodium intake is 2213 mg/day (IQR 1760–2797 mg; range 711.0–8098 mg), relative to the recommended intake of 1200 mg/day. The median excess is 1013 mg/day (84% above the recommended level) and is likewise statistically significant (*p* < 0.0001).

In children aged 5–6 years, the median sodium intake reaches 2435 mg/day (IQR 2002–3358 mg; range 723.6–6381 mg), with the same recommended intake of 1200 mg/day. The median excess is 1235 mg/day (103% above the recommended level) and is statistically significant according to the Wilcoxon test (*p* < 0.0001) (Table 8).

According to the Kruskal–Wallis test and Dunn’s test, differences between 1–2 and 3–4 years are not significant (*p* = 0.52), whereas the 5–6-year group has substantially higher ranks of sodium intake than 1–2-year-olds (*p* = 0.0009) and 3–4-year-olds (*p* = 0.0183). The mean ranks (139.0; 158.6; 191.0) reflect an age-related increase in sodium load in the diet.

Thus, in all three age groups, there is a large and statistically significant median excess of sodium intake relative to the recommended level, most pronounced in children aged 5–6 years; at the same time, a very wide range of individual values persists.

The analysis of mineral intake based on the prevalence of deficiency revealed a striking contrast between nutrients characterized by pervasive inadequacy and those consumed in excess (Table 9).

Calcium inadequacy affected more than 94% of children across all age groups, highlighting a severe and persistent deficit that did not improve with age. In contrast, sodium intake exceeded recommended levels in more than 95% of children, with a tendency toward greater excess in older age groups. Intake of potassium, magnesium, and phosphorus also exceeded reference values in the majority of participants.

### 3.5. Association Between Parental Food Group Priorities and Actual Nutrient Intake

For the Spearman correlation analysis, we proceeded from the premise that scores on the “Parental priority in feeding” scales reflect not the actual consumption of products, but parental attitudes and priorities regarding specific food groups. To examine how closely these subjective priorities are actually related to the child’s diet, we selected as Y variables those nutrients that are physiologically and structurally most closely associated with each product group and are simultaneously used in normative documents as key markers of diet quality. In this context, Spearman correlation makes it possible to assess the monotonic relationship between ordinal scores (1–5) and continuous measures of nutrient intake without requiring normal distribution.

For the “Fats and sweets” scale, it is logical to focus on total lipid intake, total energy, and carbohydrates, since this product group makes the main contribution to the energy density of the diet, the proportion of saturated fats, and “free” sugars. Sodium and phosphorus are included as markers of consumption of industrially processed foods (snacks, confectionery, processed meats, cheeses and sausages), which often combine high levels of fat, salt, and phosphate additives. Magnesium is used as an additional marker, since some sweet drinks and snack products are fortified with this mineral; its relationship with preferences for sweets is less direct, but it allows us to capture a possible accompanying effect.

For the “Meat—fish” scale, we selected nutrients traditionally associated with this group: protein, iron, niacin, and phosphorus, as well as total energy. Meat and fish are major sources of high-quality protein and heme iron, a substantial share of niacin and phosphorus, and they shape the caloric density of the diet through their fat and protein content. Riboflavin was additionally included, as it is partially provided by meat and fish and reflects the quality of the protein–fat component of the diet. Thus, if parents truly attach high importance to meat and fish, this should be reflected in higher intake of these specific nutrients.

For the “Dairy products” scale, calcium, riboflavin, protein, phosphorus, and lipids were chosen as the main markers. Dairy products are a key source of calcium and an important contributor of riboflavin, high-quality protein, and phosphorus in children; some dairy products (especially whole milk, cream, cheeses) also provide a substantial share of dietary fat. Therefore, these indicators are the most sensitive to changes in the proportion of milk and dairy products in the diet and make it possible to test whether high scores on the “dairy products” scale correspond to real improvements in calcium and protein adequacy.

The “Vegetables—fruits” scale was compared with intake of dietary fiber, vitamin C, potassium, and magnesium, and, optionally, with total carbohydrates. Vegetables and fruits are the main source of dietary fiber, vitamin C, and a substantial share of potassium and magnesium; therefore, these indicators most adequately reflect the “plant-based block” of the diet. Additional analysis by total carbohydrate intake is possible but must be interpreted with caution, since carbohydrates also come from bread, grains, and sweets. Thus, the selected set of nutrients allows us to evaluate whether high priorities for vegetables and fruits are accompanied by a real increase in their contribution to the vitamin–mineral profile of the diet.

Finally, for the “Bread and potatoes” scale, it is logical to use total carbohydrates, energy value, sodium, phosphorus, and dietary fiber. Bread and flour-based products, as well as potatoes and grains, are the main source of starch and a substantial share of daily energy in children; bread also serves as an important source of sodium (due to added salt in baking) and phosphorus (flour, leavening agents, technological additives). Dietary fiber is included as an additional marker that partly distinguishes the contribution of whole-grain versus refined grain products.

Overall, this selection of Y variables for each scale provides a biologically grounded and methodologically consistent test of the hypothesis that parental priorities correspond to the actual nutrient profiles of children’s diets (Table 10).

In the Spearman correlation analysis, no statistically significant associations were found between scores on the “Fats and sweets” intended restriction scale and daily intake of total lipids, energy, carbohydrates, sodium, phosphorus, or magnesium (for all pairs |r| < 0.07, *p* > 0.20).

For the “Meat and fish” scale, the ranks of the perceived importance of these products in parents’ views likewise showed no significant relationship with actual intake of protein, iron, niacin, phosphorus, total energy, or lipids (|r| from −0.04 to 0.10; in all cases *p* > 0.06). High or low ratings of the importance of meat and fish did not reflect the real level of their consumption by children.

For the “Dairy products” scale, Spearman coefficients were small in absolute value (0.02–0.07), confidence intervals crossed zero, and *p* values in all cases exceeded 0.22. The slight positive trend for lipids, energy, protein, and riboflavin was not statistically confirmed. Parental preferences regarding dairy products are almost not reflected in the actual structure of the child’s daily diet. Similarly, for the “Vegetables and fruits” scale, no significant associations were found with intake of dietary fiber, vitamin C, potassium, magnesium, total energy, or carbohydrates (|r| from −0.09 to 0.10; minimum *p* = 0.076 for vitamin C). Higher ratings of the importance of vegetables and fruits were not accompanied by a statistically significant increase in their consumption by children. For the “Bread and potatoes” scale, the ranks of the importance of bread and potatoes in parents’ views did not correlate with actual intake of dietary fiber, energy, carbohydrates, phosphorus, or sodium (|r| from −0.04 to 0.06; *p* > 0.26). Taken together, the results show that, across all five scales, parental declarative priorities regarding major food groups are weakly and non-significantly related to the actual nutrient profiles of children’s diets. This underscores a gap between parental attitudes and the child’s actual diet and is consistent with findings from international studies (Table 4).

## 4. Discussion

- Parental dietary priorities

Across all age subgroups, parents reported very strict control of fats and sweets: scores on this scale were close to the upper end, and we did not observe statistically meaningful age differences. Importantly, for this domain higher scores reflect stronger intended restriction (i.e., “should be minimized”), whereas lower scores reflect a tendency to allow a larger share in the diet (i.e., “should predominate”); this direction should be kept in mind when interpreting group patterns. This direction should be kept in mind when interpreting age patterns and group differences. In other words, across child ages, parents tended to report a strong intention to restrict foods perceived as “unhealthy,” such as fatty and sugary items. This pattern is consistent with previous work showing that parental concern about sweets and fatty foods is a key driver of early feeding practices and is often framed as the central component of healthy eating for young children [44]. At the same time, the near-ceiling distribution on this scale means that variability is limited by design, which mechanically constrains observable correlations with children’s nutrient intakes and must be considered when interpreting essentially null associations.

In contrast, meat and fish are perceived as moderately important but not truly priority food groups. Response distributions are very similar across ages, and we did not observe systematic age-related shifts. Thus, the perceived contribution of animal-source foods to a child’s diet appears relatively stable and does not increase as children grow older. Comparable findings have been reported elsewhere, suggesting that animal-source foods are not always framed as central in early feeding decisions and may receive less consistent emphasis than staple foods [45]. This supports the notion that meat products occupy a relatively low priority in the eyes of many parents.

Attitudes toward dairy products show a more complex age pattern. In the youngest subgroup, dairy tends to be rated less strongly as a top-priority food group compared with older children. With age, dairy tends to move toward more priority-oriented ratings and then stabilizes in the middle and older preschool years, suggesting an initial underestimation of milk and dairy products at 1–2 years followed by a “catch-up” re-evaluation later on. Age-related shifts in dairy-related patterns have also been reported elsewhere; importantly, changes in perceived importance do not necessarily translate into higher intake, and in some cohorts the proportional contribution of milk and dairy products declined with age and remained below recommended levels [46].

Perceived importance of vegetables and fruits, on average, corresponds to “moderate” rather than “high” priority. Median scores lie around the middle of the scale, with no clear movement toward either maximal priority or neglect. We observed a modest temporary dip in younger preschoolers with partial recovery in older children, but no statistically robust between-group differences. This pattern suggests fluctuating perceptions rather than a structural age gradient and suggests that vegetables and fruits may be perceived as important, yet they do not appear to be prioritized as consistently as the restriction of “unhealthy” foods. Several studies have shown that even when parents verbally acknowledge the benefits of vegetables and fruits, these groups frequently receive lower practical priority than avoiding “bad foods,” contributing to a real shortfall of vegetables and fruits in children’s diets [47].

By contrast, bread and potatoes are consistently viewed as a stable and important foundation of the diet across all age groups. Their priority remains high and does not demonstrate substantial age-related changes, indicating that starchy staples are widely perceived as a stable core component of the child’s diet. This is compatible with data from Dutch daycare settings, where bread and other cereal products, alongside dairy, formed one of the main energy sources in the diets of young children [48].

Taken together, parental attitudes in this urban Kazakhstani sample are characterized by strict control of potentially “unhealthy” foods (fats and sweets), a consistently high role for starchy staples (bread and potatoes), only moderate prioritization of vegetables, fruits, and protein-rich foods, and an underestimation of dairy products in the youngest children. This combination fits well with evidence that parental attitudes often diverge from the actual structure of preschool children’s diets [49,50]. In our data, Spearman correlations between parental priority scores and children’s nutrient intakes on the recalled day were weak and non-significant, suggesting that declared intentions may not consistently translate into more nutritionally adequate one-day intakes.

- Macronutrient profile of the diets

The macronutrient composition of the recalled diets shows a heterogeneous and age-specific pattern. For protein, the youngest children tended to have median intakes above national recommendations on the recalled day, whereas in the middle age subgroup a clear deficit emerged, with medians below reference values and substantial interindividual variability: some children markedly under-consumed protein, while others exceeded recommendations. In older preschoolers, median protein intake moved closer to the reference and fell only slightly short of it, suggesting a partial normalization with age. Significant between-group differences were observed mainly between the youngest and oldest children, consistent with a gradual shift from relative excess toward a more balanced, albeit not fully recommendation-concordant, intake. Similar age-dependent patterns—with excess protein intake in younger children, pronounced heterogeneity in preschoolers, and variability across populations—have been described in Polish and Kosovar cohorts [51,52].

For fats, the picture is more concerning. In all three age subgroups, median fat intake on the recalled day was below national recommendations, and this deficit was statistically evident. The shortfall was most pronounced in the youngest children and younger preschoolers and only partially attenuated among older preschoolers. Despite a stepwise increase in fat intake with age, the overall distribution of one-day intakes remained shifted below the recommended level, with wide variation from very low to very high fat saturation. These findings align with reports from other regions where preschool children often show substantial disturbances in fat quantity and quality, including an imbalanced fatty-acid profile and deviations from guideline targets [53].

Dietary fiber intake showed the most severe and consistent shortfall. Median values in all age groups were substantially and significantly below recommendations, and between-group comparisons did not reveal meaningful differences. As children grew older, recommended intakes increased, whereas actual intake remained at a roughly similar absolute level, resulting in a widening relative gap. This pattern indicates a population-wide deficit of fiber-rich foods—vegetables, fruits, legumes, and whole grains—in preschool diets. Comparable deficits have been reported in Polish preschoolers from Piła, where low fiber intake coincided with high sucrose and saturated-fat intake, pointing to insufficient inclusion of vegetables, fruits, and fiber-rich foods [54]. Longitudinal data from the Melbourne InFANT program likewise showed that more than half of children followed a persistently “low” fiber trajectory, with fewer than half achieving adequate intake at 18 and 60 months despite rising requirements [55].

For carbohydrates, the youngest subgroup showed median intake slightly above the reference on the recalled day, whereas younger preschoolers demonstrated a statistically significant deficit relative to recommended levels. In older preschoolers, carbohydrate intake increased and narrowed, but did not close, the gap with recommendations. Across all age groups, the distribution was extremely wide, ranging from very low to very high carbohydrate diets.

Total energy intake displayed a similar age asymmetry. Among the youngest children, median energy intake on the recalled day corresponded approximately to national recommendations, whereas younger preschoolers showed a statistically significant energy deficit. Older preschoolers partially compensated this deficit, but median caloric intake remained below target levels. Within each age group, one-day energy intakes ranged from clear underconsumption to very high energy intake, mirroring heterogeneity seen in other European and regional reports [51,56].

Taken together, the macronutrient analysis suggests that in the youngest children, average one-day protein and carbohydrate intakes are close to or slightly above recommendations, whereas by the middle preschool years a systemic deficit of energy, protein, fats, and carbohydrates emerges. By older preschool age, some indicators shift toward reference values, but deficits in fats, dietary fiber, and overall caloric intake persist, and a sizeable fraction of children remain below recommended levels on the recalled day. These prevalence-based patterns are important from a public-health perspective, as they indicate that even when age-specific medians approach targets, a substantial proportion of preschoolers experience inadequate macronutrient intake.

- Vitamin and mineral adequacy

The vitamin profile is complex and nutrient-specific. For thiamine, median intakes in the youngest and oldest subgroups on the recalled day were close to recommended values, with no statistically significant deviations, whereas middle preschoolers showed a clear median shortfall. Between-group comparisons indicated that older preschoolers had higher ranks of thiamine intake than both younger subgroups, suggesting age-related improvement in thiamine adequacy. This pattern is broadly consistent with data from other settings, where B-vitamin adequacy tends to improve with age in the context of diversified diets, even though deficiencies remain in specific nutrients such as vitamin D or vitamin A [57,58].

Riboflavin intake appeared more favorable: in all age groups, median intakes not only reached but moderately exceeded recommended values, and this excess was statistically significant. Between-group differences were small, so preschoolers as a whole can be characterized by a relatively stable, modest surplus of riboflavin on the recalled day, likely reflecting regular consumption of milk, dairy products, and fortified cereals, as reported in national data from the United Kingdom [59]. At the same time, wide individual ranges indicate that some children still have suboptimal riboflavin intake, whereas others consume very high amounts.

For niacin (vitamin PP), median intakes were below recommendations at all ages, with the most robust deficit observed in middle preschoolers. Younger and older preschoolers had medians closer to the reference, but the overall trend toward insufficient intake remained, and interindividual variability was large. Similar tendencies toward inadequate niacin and other B-vitamin intakes have been documented in Kosovar preschoolers [60].

Vitamin C showed the most unfavorable profile. In all three age groups, median vitamin C intake on the recalled day was approximately half of the recommended level, and between-group differences were negligible. Within each subgroup, intakes ranged from nearly zero to very high values, but overall, the distribution of one-day intakes was clearly shifted below recommended levels. These findings echo data from Egypt and other Eastern Mediterranean countries, where low vegetable and fruit consumption leads to systemic vitamin C deficiency across multiple child and adolescent age groups [61,62]. From a prevalence standpoint, vitamin C stands out as a population-wide concern in our sample, as the majority of children in each age group did not meet recommendations on the recalled day.

Mineral adequacy also shows a mixed pattern. For iron, median intakes in young children and younger preschoolers were significantly below recommended levels, indicating a high risk of dietary shortfalls. In older preschoolers, median iron intake came close to the reference, and the group-level deficit was no longer statistically apparent, but variability remained large, with some children consuming very low and others very high amounts. This combination of persistent risk in younger ages and partial normalization later on is compatible with global estimates suggesting that iron inadequacy remains a key concern in early childhood in many low- and middle-income contexts [63,64].

In contrast, other minerals demonstrated a tendency toward excess relative to national reference values. On the recalled day, intakes of potassium, magnesium, sodium, and phosphorus were frequently well above recommended levels, and this pattern was consistent across age groups despite wide individual variability. In particular, median sodium intake clearly exceeded recommended levels, indicating a structurally high salt content of preschool diets. At the same time, calcium intake was substantially and consistently below recommended values in all age groups, and this deficit did not diminish with age. Thus, preschool children in this urban Kazakhstani sample are simultaneously exposed to insufficient calcium and iron, low vitamin C and dietary fiber, and excessive sodium and phosphorus, a combination that is broadly compatible with a nutrient-imbalanced dietary pattern dominated by refined staples and processed foods [11,56].

- Integrated conclusions and interpretation

When parental attitudes and children’s one-day dietary intakes are considered together, a contradictory pattern emerges. On the one hand, many parents endorse strong control of overtly “unhealthy” foods and express high concern about fats and sweets. On the other hand, the actual composition of children’s diets on the recalled day shows systematic shortfalls in key nutrients—energy, fats, dietary fiber, calcium, iron, vitamin C, and, intermittently, thiamine and niacin—against a background of excessive sodium and phosphorus. This mismatch between declared oncern about healthy eating and the nutrient profile of children’s diets is consistent with broader evidence that parental perceptions of diet quality often do not align with objective dietary indicators [65,66].

The middle preschool subgroup appears particularly vulnerable: it is at this stage that a clear deficit of energy and several macronutrients (protein, fats, carbohydrates) is most pronounced, while vitamin and mineral inadequacies remain common. This may reflect the combined influence of preschool institutional menus and home foods in a period when children are increasingly exposed to organized catering but still depend heavily on parental decisions. Similar dual contributions of institutional and home food to nutritional risks have been described in studies of daycare and kindergarten meals, where school or daycare menus and home diets each supply roughly half of total energy intake but the combined pattern remains suboptimal [67,68].

In the youngest children, one-day protein and carbohydrate intakes often meet or exceed recommendations, and median energy intake is close to target levels, yet dietary fiber, vitamin C, calcium, and iron are already insufficient for many children. Older preschoolers show partial improvement in protein, thiamine, niacin, and iron intakes, but persistent shortfalls in fats, dietary fiber, calcium, and vitamin C, together with high sodium and phosphorus. From a life-course perspective, these findings suggest that the preschool years in urban Kazakhstan represent a critical window in which “double-edged” dietary patterns are consolidated: overt restriction of sweets and visible fats coexists with insufficient provision of nutrient-dense plant foods and calcium-rich products, and with high exposure to salt-rich, processed foods.

For public health practice, this implies that interventions should not focus solely on reinforcing parental concern about sweets and fatty foods, which is already high, but rather on reshaping the overall structure of preschool diets. Priority targets include increasing the availability and acceptability of vegetables, fruits, whole grains, and dairy products (particularly in the youngest subgroup), reducing sodium from processed foods and added salt, and improving the quality and balance of fats. Because stated parental priorities showed only weak associations with children’s one-day intakes, effective strategies will likely need to combine parental education with structural measures in preschools and kindergartens, such as menu standards, procurement policies, and supportive feeding practices, in order to translate intentions into sustained dietary improvements at the population level.

## 5. Conclusions

In this sample of urban children aged 1–6 years in Central Kazakhstan, we documented a pronounced mismatch between parents’ stated dietary priorities and children’s actual nutrient intake. Despite near-universal emphasis on limiting fats and sweets and a consistently high priority assigned to bread and potatoes, children’s diets were characterized by systematic shortfalls in several key nutrients and persistent excess sodium.

The macronutrient profile was clearly age-specific. In the youngest children, diets showed relative excesses of protein and carbohydrates combined with a marked deficit of fats. Among 3–4-year-olds, multiple deficits converged—energy, protein, fats, carbohydrates, thiamine, niacin, and iron—while in 5–6-year-olds some indicators moved closer to reference values, but intakes of fat and total energy remained suboptimal. Thus, younger preschoolers who are just entering organized feeding appear to be the most vulnerable group in terms of overall nutrient adequacy.

Diet quality was consistently unfavorable with respect to dietary fiber and vitamin C, with median intakes significantly below national recommendations in all age subgroups. Iron intake showed significant median shortfalls in the younger age groups, with values approaching the reference level only by 5–6 years. Thiamine and niacin showed intermittent deficits, especially at 3–4 years, against a background of moderately elevated riboflavin intake, indicating an imbalanced vitamin B profile rather than uniformly adequate coverage.

Across all five parental-priority scales, correlations with nutrient intakes were weak and non-significant, indicating that declarative concern about healthy eating only minimally translates into the nutrient profile of children’s diets. This perception–intake gap coexists with very wide interindividual variability—from pronounced deficiencies to near excess—and points to deep heterogeneity of dietary situations among urban families.

In addition, for some items such as limiting fats and sweets, parental priority scores showed pronounced ceiling effects, with most respondents selecting the highest category. This restricted range likely attenuated any underlying associations with children’s intakes and may partly explain the weak or null correlations observed for these food groups.

Taken together, these findings argue for comprehensive correction of preschoolers’ diets at both the family and institutional levels, with particular attention to children aged 3–4 years. Priority directions include reorienting parent education programs from an almost exclusive focus on “avoiding unhealthy foods” toward ensuring adequate intake of fats, dietary fiber, vitamin C, calcium, and iron-rich foods; revising preschool menus in light of the identified deficits, especially for younger preschoolers starting kindergarten; and targeted counseling and follow-up for families in which dietary data reveal multiple nutrient shortfalls. Even in an urban context with high declared concern about healthy eating, our data show that preschool children’s diets in Central Kazakhstan remain far from optimal and require coordinated public health action rather than only individual-level advice.

## 6. Limitations

First, the total number of families initially approached and the detailed reasons for non-participation at the recruitment stage were not systematically recorded in the original field logs. As a result, a formal response rate cannot be calculated and the potential for selection bias cannot be fully quantified.

Second, dietary intake was assessed using a single, non-consecutive 24-h recall per child. Although we applied a standardized multiple-pass protocol with trained interviewers and portion size aids, a single recall cannot fully capture day-to-day variability in intake, especially in preschool children. Therefore, our analyses describe the distribution of one-day intakes relative to national reference values rather than children’s usual intake, and we were not able to quantify the prevalence of nutrient inadequacy in a probabilistic sense. In addition, we did not administer a complementary food frequency questionnaire or repeated recalls, so we could not characterize longer-term eating patterns, the usual frequency of consumption of specific food groups, or the intake of foods eaten only occasionally. This limitation is particularly relevant in young children, whose diets may vary substantially from day to day, and implies that our findings should be interpreted as reflecting one-day intake distributions rather than fully capturing habitual dietary patterns.

Third, for children attending kindergarten, intakes at institutional meals were reconstructed from standard production cards and caregivers’ semi-quantitative reports (e.g., “ate all”, “about half”, “only tasted”). This approach may better reflect the foods offered than the foods actually consumed and may therefore introduce measurement error in nutrient intake estimates, particularly in 3–4-year-olds who frequently leave part of the portion uneaten. No formal validation of caregiver reports against plate-waste assessment or direct observation was performed, and our findings should be interpreted in light of this potential source of bias.

Fourth, correlations between parental dietary priorities and children’s intakes were computed in the pooled 1–6-year sample without adjustment for key confounders, including age group and city of residence. These associations may therefore be partly driven by residual confounding and should be interpreted with caution as exploratory, hypothesis-generating findings rather than adjusted causal estimates. Although basic sociodemographic variables (parental education and self-reported household income) were collected, the present analysis did not formally model socioeconomic gradients in children’s diets, so residual socioeconomic confounding cannot be excluded.

Fifth, the parental priority scales for some domains (particularly limiting fats and sweets) exhibited marked ceiling effects, with highly skewed distributions toward the most “healthy” responses. This limited variability may have mechanically reduced observable correlations with dietary intake and suggests that our null findings for these items should be interpreted with caution.

Finally, we examined a broad set of nutrients using multiple one-sample tests, which increases the risk of type I error (false-positive findings). The analyses should therefore be interpreted as exploratory with respect to individual micronutrients, and our conclusions focus primarily on a predefined set of key nutrients (energy, total fat, dietary fiber, calcium, iron, vitamin C and sodium) and on the magnitude and consistency of one-day shortfalls or excesses relative to national reference values.

## Figures and Tables

**Table 1:** Distribution of participants by age group and city.

City	1–2 Years, *n* (%)	3–4 Years, *n* (%)	5–6 Years, *n* (%)	Total, *n*
Astana	53 (33.8%)	76 (48.4%)	28 (17.8%)	157
Karaganda	65 (19.2%)	217 (64.2%)	106 (31.4%)	338

**Table 2:** Sociodemographic characteristics of children aged 1–6 years included in the analytic sample (*n* = 390).

Category	*n*	% of Total
Age group		
1–2 years	118	30.3
3–4 years	136	34.9
5–6 years	136	34.9
Sex		
Boys	187	47.9
Girls	203	52.1
City of residence		
Astana	163	41.8
Karaganda	227	58.2
Main daytime setting		
Home (with mother/caregiver, etc.)	155	39.7
Kindergarten	235	60.3
Parental marital status		
Married or cohabiting	344	88.2
Divorced or separated	26	6.7
Never married	20	5.1
Widowed	0	0.0
Maternal education		
Below secondary	0	0.0
Secondary	33	8.5
Vocational/technical	98	25.1
Higher (including ≥3 years of university)	259	66.4
Household income (KZT/month)		
<50,000	0	0.0
50,000–100,000	140	35.9
100,000–200,000	132	33.8
>200,000	118	30.3

**Table 3:** Distribution of parental priority scores across food categories by age group (%).

Age Group	Food Category	Score 1 (%)	Score 2 (%)	Score 3 (%)	Score 4 (%)	Score 5 (%)
1–2 years	Fats and sweets	0.9	0.0	9.6	8.7	80.9
1–2 years	Meat and fish products	40.9	26.1	27.8	3.5	1.7
1–2 years	Milk and dairy products	35.7	47.0	16.5	0.9	0.0
1–2 years	Vegetables and fruits	20.9	16.5	10.4	51.3	0.9
1–2 years	Cereals, bread, and potatoes	3.5	12.2	33.0	32.2	19.1
3–4 years	Fats and sweets	1.4	0.7	5.5	7.2	85.3
3–4 years	Meat and fish products	32.8	29.4	20.1	14.7	3.1
3–4 years	Milk and dairy products	36.9	23.9	29.4	9.2	0.7
3–4 years	Vegetables and fruits	22.9	24.9	26.6	25.3	0.3
3–4 years	Cereals, bread, and potatoes	3.8	22.9	21.2	44.0	8.2
5–6 years	Fats and sweets	4.5	3.0	5.3	9.0	78.2
5–6 years	Meat and fish products	34.6	27.1	24.1	10.5	3.8
5–6 years	Milk and dairy products	31.6	24.8	33.1	6.8	3.8
5–6 years	Vegetables and fruits	16.5	25.6	23.3	30.1	4.5
5–6 years	Cereals, bread, and potatoes	7.5	21.1	18.8	43.6	9.0

Note. Scores indicate parental priorities for each food category using a five-point scale: Score 1—“should predominate in the diet”, Score 2—“important, preferably consumed often”, Score 3—“neutral/moderate”, Score 4—“should be limited”, Score 5—“should be minimized in the diet”.

**Table 4:** Characteristics of actual dietary intake (macronutrients) in the study groups.

Indicator	Children 1–2 Years Me (25–75%)	Children 3–4 Years Me (25–75%)	Children 5–6 Years Me (25–75%)
Protein (g)	35.80 (21.73–53.41)	38.87 (29.63–58.25)	43.50 (32.62–58.90)
Lipids (g)	32.15 (22.49–49.05)	42.16 (28.89–57.40)	53.43 (33.69–75.22)
Carbohydrates (g)	169.4 (130.4–237.0)	177.0 (139.7–216.8)	196.1 (162.8–242.8)
Dietary fiber (g)	9.30 (7.15–12.66)	9.87 (7.15–12.86)	10.74 (8.07–14.40)
Energy (kcal)	1170 (851.8–1648)	1299 (1064–1546)	1510 (1182–1871)

Note. Me—median; 25–75%—interquartile range (IQR; 25th–75th percentiles).

**Table 5:** Prevalence of inadequate and excessive intake of energy and macronutrients by age group (%).

Indicator	1–2 Years: % Below Recommendation	1–2 Years: % Above Recommendation	3–4 Years: % Below Recommendation	3–4 Years: % Above Recommendation	5–6 Years: % Below Recommendation	5–6 Years: % Above Recommendation
Energy intake, kcal/day	51.5	48.5	80.9	19.1	70.7	29.3
Protein, g/day	39.7	60.3	66.2	33.8	60.2	39.8
Fat, g/day	75.0	25.0	76.1	23.9	60.9	39.1
Carbohydrates, g/day	47.1	52.9	81.2	18.8	80.5	19.5
Dietary fiber, g/day	91.2	8.8	99.7	0.3	99.2	0.8

**Table 6:** Characteristics of vitamin intake in the study groups.

Indicator	Children 1–2 Years Me (25–75%)	Children 3–4 Years Me (25–75%)	Children 5–6 Years Me (25–75%)
Vitamin B1, mg	0.467 (0.333–0.687)	0.499 (0.382–0.692)	0.613 (0.444–0.788)
Vitamin B2, mg	0.6520 (0.5120–0.9638)	0.6590 (0.5120–0.9025)	0.7015 (0.5548–0.9440)
Vitamin PP, mg	4.99 (3.079–8.831)	6.25 (4.325–8.700)	7.23 (4.708–9.770)
Vitamin C, mg	16.27 (9.05–23.02)	15.75 (7.29–27.60)	17.81 (7.70–31.82)

**Table 7:** Prevalence of inadequate and excessive vitamin intake by age group (%).

Indicator	1–2 Years: % Below Recommendation	1–2 Years: % Above Recommendation	3–4 Years: % Below Recommendation	3–4 Years: % Above Recommendation	5–6 Years: % Below Recommendation	5–6 Years: % Above Recommendation
B_1_ (thiamine), mg/day	58.8	41.2	54.9	44.4	48.9	51.1
B_2_ (riboflavin), mg/day	29.4	70.6	39.2	60.4	32.3	66.9
PP (niacin), mg/day	55.9	44.1	68.3	31.7	58.6	41.4
C, mg/day	91.2	8.8	74.1	25.9	72.9	27.1

**Table 8:** Characteristics of mineral intake in the studied groups.

Indicator	Children 1–2 Years Me (25–75%)	Children 3–4 Years Me (25–75%)	Children 5–6 Years Me (25–75%)
Iron, mg	7.17 (4.56–10.06)	8.18 (6.81–10.36)	9.73 (7.76–12.19)
Potassium, mg	1244 (864.4–1821)	1285 (944.4–1728)	1368 (1008–1932)
Calcium, mg	360.7 (250.5–533.5)	372.1 (269.8–479.6)	392.9 (289.4–523.1)
Magnesium, mg	142.9 (100.1–207.7)	158.5 (119.2–187.9)	164.3 (121.3–232.5)
Sodium, mg	1976 (1760–2810)	2213 (1760–2797)	2435 (2002–3358)
Phosphorus, mg	623.1 (455.3–823.9)	647.5 (525.8–807.6)	721.4 (575.7–915.1)

**Table 9:** Prevalence of inadequate and excessive mineral intake by age group (%).

Indicator	1–2 Years: % Below Recommendation	1–2 Years: % Above Recommendation	3–4 Years: % Below Recommendation	3–4 Years: % Above Recommendation	5–6 Years: % Below Recommendation	5–6 Years: % Above Recommendation
Calcium, mg/day	96.9	3.1	95.2	4.8	94.0	6.0
Iron, mg/day	72.3	27.7	63.8	35.5	53.4	46.6
Potassium, mg/day	0.0	100.0	1.7	98.3	1.5	98.5
Magnesium, mg/day	21.5	76.9	2.7	97.3	9.8	90.2
Sodium, mg/day	3.1	96.9	4.4	95.6	1.5	98.5
Phosphorus, mg/day	9.2	90.8	18.1	81.9	15.0	85.0

**Table 10:** Spearman correlation coefficients between parental priority scale scores and daily nutrient intake in children aged 1–6 years.

Scale (X)/X–Y Pair	Spearman r	95% CI for r	*p* (Two-Sided)
Fats and sweets			
lipids (g/day)	−0.0356	−0.1458 to 0.0755	0.5181
energy (kcal/day)	0.0326	−0.0785 to 0.1429	0.5541
carbohydrates (g/day)	0.0675	−0.0436 to 0.1770	0.2200
sodium (mg/day)	−0.0044	−0.1152 to 0.1064	0.9360
phosphorus (mg/day)	0.0286	−0.0824 to 0.1390	0.6031
magnesium (mg/day)	−0.0444	−0.1544 to 0.0668	0.4206
Meat and fish			
lipids (g/day)	0.0839	−0.0272 to 0.1929	0.1271
energy (kcal/day)	0.1029	−0.0080 to 0.2113	0.0611
protein (g/day)	0.0075	−0.1034 to 0.1182	0.8914
iron (mg/day)	0.0491	−0.0621 to 0.1590	0.3727
phosphorus (mg/day)	−0.0102	−0.1208 to 0.1007	0.8535
niacin (PP), mg/day	−0.0407	−0.1508 to 0.0704	0.4595
riboflavin (B_2_), mg/day	−0.0180	−0.1290 to 0.0925	0.7371
Dairy products			
lipids (g/day)	0.05064	−0.06049 to 0.1605	0.3576
energy (kcal/day)	0.05371	−0.05742 to 0.1635	0.3292
protein (g/day)	0.06679	−0.04433 to 0.1763	0.2248
calcium (mg/day)	−0.01700	−0.1276 to 0.09397	0.7576
phosphorus (mg/day)	−0.01780	−0.1283 to 0.09318	0.7466
riboflavin (mg/day)	0.04574	−0.06539 to 0.1557	0.4061
Vegetables and fruits			
dietary fiber (g/day)	−0.0815	−0.1906 to 0.0295	0.1383
energy (kcal/day)	−0.0876	−0.1964 to 0.0235	0.1113
carbohydrates (g/day)	−0.0774	−0.1866 to 0.0337	0.1595
vitamin C (mg/day)	0.0975	−0.0135 to 0.2060	0.0761
potassium (mg/day)	−0.0232	−0.1336 to 0.0879	0.6742
magnesium (mg/day)	−0.0711	−0.1805 to 0.0400	0.1960
Bread and potatoes			
dietary fiber (g/day)	0.010	−0.1009 to 0.1206	0.8564
energy (kcal/day)	0.044	−0.0672 to 0.1540	0.4246
carbohydrates (g/day)	−0.039	−0.1490 to 0.0723	0.4805
phosphorus (mg/day)	0.062	−0.0494 to 0.1714	0.2618
sodium (mg/day)	−0.023	−0.1336 to 0.0879	0.6746

## Data Availability

The data presented in this study are available upon reasonable request from the corresponding author.

## Supplementary Materials

The following supporting information can be downloaded at: https://www.mdpi.com/article/10.3390/ijerph23010109/s1, File S1: 24-Hour Multiple-Pass Dietary Recall Questionnaire (children) with Blank Informed Consent.

## Abbreviations

AI	Adequate Intake
AMDR	Acceptable Macronutrient Distribution Range(s)
CACFP	Child and Adult Care Food Program
CI	Confidence Interval
DGA	Dietary Guidelines for Americans
DRI	Dietary Reference Intake(s)
ECEC	Early Childhood Education and Care
EsNuPI	Estudio Nutricional en Población Infantil
FITS	Feeding Infants and Toddlers Study
IQR	Interquartile Range
NDNS	National Diet and Nutrition Survey (UK)
NPNS	National Pre-School Nutrition Survey (Ireland)
PP	Vitamin PP (niacin)
RDA	Recommended Dietary Allowance
RR	Relative Risk
SACN	Scientific Advisory Committee on Nutrition (UK)
SD	Standard Deviation
UNICEF	United Nations Children’s Fund
