# Supplementary material for: Parental Perceptions of Healthy Eating and Actual Nutrient Intake: Analysis of the Nutritional Status of Children Aged 1–6 Years in Urban Areas of Central Kazakhstan
Source: Int J Environ Res Public Health. 2026 Jan 15;23(1):109. doi: 10.3390/ijerph23010109 (PMC12841158; doi:10.3390/ijerph23010109)
Supplement: Supplementary file 1 [file ijerph-23-00109-s001.zip › ijerph-4058292-supplementary.pdf]

Ministry of Health of the Republic of Kazakhstan
NAO KMU

Questionnaire for Study Participant

“Nutritional Profile of Children in the Karaganda Region”
ASSESSMENT
OF ACTUAL NUTRITIONAL STATUS
OF THE CHILD POPULATION

Registration number ______________________________

Province/Region of residence______________________

City/Town____________________________________

District_______________________________________

Karaganda 2023

INFORMATION FOR THE STUDY PARTICIPANT

Dear respondent! We invite you to take part in this survey.
The aim of this study is to optimize children's nutrition as one of the key approaches to improving the health status of the child population of the Karaganda region.
Lead executor: NAO Karaganda Medical University.
The assessment of actual dietary intake will be conducted using standardized international questionnaires.

The study is conducted with the respondent's voluntary informed consent; no financial compensation is provided for participation. All data obtained from respondents are confidential.

Dear respondent, please answer the questions in the questionnaire. If you agree to participate in this study, you need to read and sign the Informed Consent form.
Thank you for your cooperation!

INFORMED CONSENT OF THE RESPONDENT

I have been informed in detail and in an accessible manner about the scientific study being conducted. I agree to participate voluntarily and without compensation in this scientific study and to fill out the questionnaires provided to me independently or to answer the questions asked of me.

It has been clearly explained to me that the information obtained as a result of the survey is confidential. During processing, the information will be de-identified (it will be impossible to identify the study participant) and used exclusively for scientific purposes.

Full name: ____
Address: ____
Contact phone: ____
(This information is necessary for the organizers to clarify your responses to the questionnaire if needed; it is confidential and will not be used elsewhere.)
Date: " ______ " ____________________ 2019
Signature: __________________

ASSESSMENT
OF ACTUAL NUTRITIONAL STATUS
OF THE CHILD POPULATION

1.1 Place of residence (specify city, region, village, etc.) ________________________
1.2 Date of completing the questionnaire ____________________________________
1.3 Child's date of birth ____
1.31 Parents' age Mother ________________ Father ____________________
1.4 Child's sex Male Female (circle or underline)
1.5 Nationality __________________________________
1.6 Parents' marital status
• Married or living in a civil union
• Divorced or living separately
• Never married
• Widowed
1.7 Parents' education level
• Below secondary
• Secondary
• Secondary specialized/vocational
• Higher (including incomplete higher education if 3+ years)
1.8 What is your household's monetary income? (Total, including all income: wages, pensions, stipends, benefits and other payments)
• less than 50,000 tenge
• 50,000–100,000 tenge
• 100,000–200,000 tenge
• more than 200,000 tenge
1.9 Number of household members (those who live with you)
• I live alone
• 2 people
• 3 people
• 4 people
• 5 people
• if more, specify how many _______________________________
1.91 Of them, children:
• 1 child
• 2 children
• 3 children
• 4 children
• 5 children
• More than 5 (specify how many) _________________________
1.10 Your child:
• Stays at home (with mother, nanny, etc.)
• Attends kindergarten
• Attends school
• Other (specify) ______________________
1.11 How do you get from home to the healthcare facility?
• On foot
• By personal car
• By public transport
1.12 How long does it take you to get from home to the healthcare facility?
• up to 15 minutes
• 15 to 30 minutes

• 30 to 45 minutes
• more than 45 minutes

2. Anthropometry

2.1 What is your weight? Mother ____________ kg   Father ____________ kg
2.11 What is your child's weight? ______________________________ kg
2.2 What is your height? Mother ____________ cm   Father ____________ cm
2.21 What is your child's height? ______________________________ cm

3. Awareness of healthy eating principles

3.1 What share of the household income is spent on the child's food (%) :
3.2 What type of feeding did your child receive:
• breastfeeding – 1
• formula feeding – 2
• mixed – 3
3.3 How long was your child on exclusive (breast) feeding:
• up to 40 days – 1
• up to 3 months – 2
• up to 6 months – 3
• up to 9 months – 4
• up to 1 year – 5
• up to 1.5 years – 6
• up to 2 years – 7
3.4 Reason for discontinuation of breastfeeding:
• lack of breast milk – 1
• child refused the breast – 2
• child's illness – 3
• mother's illness – 4
• other reasons – 5 (specify)
3.5 At what age was the first complementary food or supplementation introduced:
• from 4 weeks – 1
• from 2 months – 2
• from 3 months – 3
• from 6 months – 4
• from 9 months – 5
• from 10–12 months – 6
• older than 1 year – 7
3.6 What was introduced as the first complementary food or supplementation:
• fruit juice – 1
• fruit puree – 2
• cottage cheese/curd – 3
• egg yolk – 4
• vegetable puree – 5
• milk porridge (specify which) _________________ 6
• meat puree – 7
• kefir, ayran – 8
• milk – 9
• rusks, cookies – 10
• other products (specify) _____ 11
3.7 At what age was the second complementary food or supplementation introduced:
• from 4 weeks – 1
• from 2 months – 2
• from 3 months – 3
• from 6 months – 4

• from 9 months – 5
• from 10–12 months – 6
• older than 1 year – 7
3.8 What was introduced as the second complementary food or supplementation:
• fruit juice – 1
• fruit puree – 2
• cottage cheese/curd – 3
• egg yolk – 4
• vegetable puree – 5
• milk porridge (specify which) ________________ 6
• meat puree – 7
• kefir, ayran – 8
• milk – 9
• rusks, cookies – 10
• other products (specify) ___________________ 11
3.9 At what age was the third complementary food or supplementation introduced:
• from 4 weeks – 1
• from 2 months – 2
• from 3 months – 3
• from 6 months – 4
• from 9 months – 5
• from 10–12 months – 6
• older than 1 year – 7
3.10 What was introduced as the third complementary food or supplementation:
• fruit juice – 1
• fruit puree – 2
• cottage cheese/curd – 3
• egg yolk – 4
• vegetable puree – 5
• milk porridge (specify which) ________________ 6
• meat puree – 7
• kefir, ayran – 8
• milk – 9
• rusks, cookies – 10
• other products (specify) _________________ 11
3.11 Please indicate at what age the following foods were introduced into your child's diet:
• fruit juice _________________________________
• fruit puree ________________________________
• cottage cheese/curd ________________________
• egg yolk __________________________________
• vegetable puree ___________________________
• milk porridge (specify which) ______________
• meat puree ________________________________
• kefir, ayran ________________________________
• milk ______________________________________
• rusks, cookies _____________________________
3.12 Which product did you use for artificial feeding of your child:
• infant formula (specify which) __________________________ 1
• milk (specify which) _________________________________ 2
• milk porridge (specify which) _________________________ 3
• other products (specify) _____________________________ 4

3.13 In your opinion, how many grams of fresh vegetables and fruits should your child consume daily?
• Number of grams ____
• Difficult to answer
• Prefer not to answer
3.14 Which foods should predominate in your child's daily diet? Rate the food groups on a 5-point scale from 1 to 5, where 1 is the food group that should be present in the largest amount and 5 in the smallest amount.
• Fats, sweets
• Meat and fish products
• Milk and dairy products
• Vegetables, fruits
• Grains, bread, potatoes
3.15 Which milk is in your opinion the healthiest for your child?
• Low-fat or fat-free (fat content 2.5–0.5%)
• Regular milk with 3.2%, 6% or higher fat content
• It is better not to consume milk at all
3.16 Which bread is in your opinion the healthiest for your child?
• White
• Grey (mixed wheat/rye)
• Bran
• Rye
• Pastries (buns, cakes, etc.)
3.17 Which meat is in your opinion the healthiest for your child?
• Beef
• Mutton/Lamb
• Poultry
• Pork
• Fish
• Other (specify) ______________________________
3.18 Are pasta products healthy for your child?
• Yes
• No
• Healthy in limited amounts
• Difficult to answer
3.19 Is potato healthy for your child?
• Yes
• No
• Healthy in limited amounts
• Healthy when boiled
• Difficult to answer
3.20 Which salt is better to consume?
• None
• Regular
• Iodized
3.21 Please indicate the sources from which you obtain information about proper nutrition for your child:
• TV programs
• Healthcare professionals
• Books, brochures
• Relatives, acquaintances
• Other (specify) _____________________________

• I do not receive such information

4. Eating habits

4.1 How many times per day does your child eat:
• once – 1
• twice – 2
• three times – 3
• more than three times – 4

4.2 What predominates in your child’s diet:
• meat dishes – 1
• flour-based dishes – 2
• vegetable-based dishes – 3
• dairy – 4

4.3 Does your child have breakfast at set times?
• yes – 1
• no – 2

4.4 Does your child have lunch at set times?
• yes – 1
• no – 2

4.5 Does your child have dinner at set times?
• yes – 1
• no – 2

4.6 Time of first breakfast:
• Earlier than 7:00
• Between 7:00 and 9:00
• Between 9:00 and 11:00
• After 11:00
• No set time
• Does not have first breakfast

4.7 Time of second breakfast:
• Earlier than 10:00
• After 12:00
• Between 10:00 and 11:00
• Between 11:00 and 12:00
• No set time
• Does not have second breakfast

4.8 Time of lunch:
• Earlier than 12:00
• Between 12:00 and 14:00
• Between 14:00 and 16:00
• Between 16:00 and 18:00
• No set time
• Does not have lunch

4.9 Time of afternoon snack:
• Earlier than 15:00
• Between 15:00 and 17:00
• No set time
• Does not have afternoon snack

4.10 Time of dinner:
• Earlier than 18:00
• Between 18:00 and 20:00
• Between 20:00 and 22:00
• After 22:00

• No set time
• Does not have dinner
4.11 Does your child eat before sleep (within 30 minutes before bedtime)?
• Yes – 1
• No – 2
4.12 How many times a day does your child eat hot food (cooked dishes)?
• once – 1
• twice – 2
• three times – 3
• more than three times – 4
• no – 5
4.13 Consumption of first-course dishes (soups, etc.):
• every day – 1
• often (4 times a week or more) – 2
• sometimes (3 times a week or less) – 3
• no – 4
4.14 How often do you and your child consume the following products? (Put only one mark "+" for each product.)

#	Products	YOU						YOUR CHILD					
		Every day	Several times a week	Once a week	Several times a month	No more than once a month	Rarely or never	Every day	Several times a week	Once a week	Several times a month	No more than once a month	Rarely or never
1.	Milk												
2.	Kefir, ayran, yogurt, sour cream, cream												
3.	Cottage cheese/curd, irimshik, cheese												
4.	Butter												
5.	Kumys, shubat												
6.	Vegetable oil												
7.	Culinary fat, margarine												

8.	Animal fats												
9.	Meat												
10.	Sausage products												
11.	Meat offal												
12.	Canned meat and fish												
13.	Fish												
14.	Eggs												
15.	Bakery products												
16.	Pasta, homemade noodles												
17.	Flour confectionery (cakes, pastries, cookies)												
18.	Grains/cereals (rice, millet, buckwheat)												
19.	Carrot												
20.	Legumes (beans, peas, etc.)												
21.	Potatoes												
22.	Cabbage (fresh, sauerkraut)												
23.	Tomatoes, cucumbers												
24.	Other vegetables (fresh)												
25.	Melons/gourds (watermelon, melon, pumpkin)												
26.	Vegetables, canned												

27.	Fresh fruits (apples, pears)												
28.	Citrus fruits												
29.	Bananas, pineapples, etc.												
30.	Berries (fresh/canned)												
31.	Vegetable and fruit-berry juices												
32.	Sugar												
33.	Confectionery												
34.	Honey												
35.	Nuts												
36.	Sunflower seeds, halva												
37.	Mushrooms												
38.	Tea												
39.	Coffee												

5. 24-hour dietary record

Please tell us what your child ate and drank over the past 24 hours—from the time you got up in the morning until you went to bed. If your child ate or drank at night, please tell us about that as well. Please do not forget to include foods and beverages consumed outside the home. Include all types of foods and drinks your child consumed. Also indicate where your child ate.

5.1 Yesterday, your child ate:

- The same amount as usual — 1
- Less than usual — 2
- More than usual — 3
- Difficult to answer — 7
- Prefer not to answer — 9

5.2 Was yesterday's eating related to:

- Doctor's recommendations — Yes/No/Difficult to answer/Prefer not to answer (1/2/7/9)
- Following a special diet — 1/2/7/9
- Religious customs — 1/2/7/9

5.3 Please indicate what your child ate yesterday:

Meal #	Time	Place of eating	Name & composition of food, dish or beverage	Approx. quantity/volume/weight CODE

1		At home (or visiting) – 1; Food service enterprise – 2; School/kindergarten etc. – 3; Workplace – 4; Other – 5		
2		At home (or visiting) – 1; Food service enterprise – 2; School/kindergarten etc. – 3; Workplace – 4; Other – 5		
3		At home (or visiting) – 1; Food service enterprise – 2; School/kindergarten etc. – 3; Workplace – 4; Other – 5		
4		At home (or visiting) – 1; Food service enterprise – 2; School/kindergarten etc. – 3; Workplace – 4; Other – 5		
5		At home (or visiting) – 1; Food service enterprise – 2; School/kindergarten etc. – 3; Workplace – 4; Other – 5		
6		At home (or visiting) – 1; Food service enterprise – 2; School/kindergarten etc. – 3; Workplace – 4; Other – 5		
